# Single-cell transcriptomic profiling reveals liver fibrosis in colorectal cancer liver metastasis

**DOI:** 10.1038/s12276-025-01573-3

**Published:** 2025-11-14

**Authors:** Yiqiao Deng, Chengyao Guo, Xiaomeng Liu, Xin Li, Jianmei Liu, Wenjie Liu, Jinghua Chen, Zhen Huang, Yefan Zhang, Xinyu Bi, Jianjun Zhao, Jianguo Zhou, Zhiyu Li, Hongliang Wu, Baocai Xing, Qichen Chen, Hong Zhao

**Affiliations:** 1 Department of Hepatobiliary Surgery, National Cancer Center/National Clinical Research Center for Cancer/Cancer Hospital, Chinese Academy of Medical Sciences and Peking Union Medical College, Beijing, 100021 China; 2 State Key Laboratory of Molecular Oncology, National Cancer Center/National Clinical Research Center for Cancer/Cancer Hospital, Chinese Academy of Medical Sciences and Peking Union Medical College, Beijing, 100021 China; 3 Department of Anesthesiology, National Cancer Center/National Clinical Research Center for Cancer/Cancer Hospital, Chinese Academy of Medical Sciences and Peking Union Medical College, Beijing, 100021 China; 4 Key Laboratory of Carcinogenesis and Translational Research (Ministry of Education/Beijing), Hepatopancreatobiliary Surgery Department I, Peking University Cancer Hospital and Institute, Beijing, 100142 China; 5 Department of Colorectal Surgery, National Cancer Center/National Clinical Research Center for Cancer/Cancer Hospital, Chinese Academy of Medical Sciences and Peking Union Medical College, Beijing, 100021 China

**Keywords:** Genetics research, Colorectal cancer

## Abstract

Tumor fibrosis is recognized as a malignant hallmark in various solid tumors; however, the clinical importance and associated molecular characteristics of tumor fibrosis in liver metastases (LM) from colorectal cancer (CRLM) remain poorly understood. Here we show that patients with CRLM whose liver metastases (LM) exhibited tumor fibrosis (Fibrosis+ LM) had significantly worse progression-free survival (*P* = 0.025) and overall survival (*P* = 0.008). Single-cell RNA sequencing revealed that the tumor microenvironment of the Fibrosis+ LM was characterized by T cells with an exhausted phenotype, macrophages displaying a profibrotic and suppressive phenotype and fibrosis-promoting fibroblasts. Further investigation highlighted the pivotal role of VCAN_eCAF in remodeling the tumor fibrosis in the tumor microenvironment of Fibrosis+ LM, emphasizing potential targetable interactions such as *FGF23* or *FGF3*-*FGFR1*. Validation through multiplex immunohistochemistry/immunofluorescence and spatial transcriptomics supported these findings. Here we present a comprehensive single-cell atlas of tumor fibrosis in LM, revealing the intricate multicellular environment and molecular features associated with it. These insights deepen our understanding of tumor fibrosis mechanisms and inform improved clinical diagnosis and treatment strategies.

## Single-cell atlas reveals fibrosis in liver tumors

The liver is a common site for cancer spread, especially from colorectal cancer. This study looks at how fibrosis in liver metastases affects treatment and survival. Researchers studied 471 patients with liver metastases from colorectal cancer. They examined tissue samples to see how fibrosis impacts survival and used advanced techniques such as single-cell RNA sequencing to understand the tumor environment better. They found that patients with more fibrosis had worse survival rates. The study also revealed that fibrosis changes the tumor environment, making it harder for the immune system to fight cancer. This includes an increase in certain immune cells that suppress the body’s defense against tumors. The researchers suggest that targeting fibrosis could improve treatment outcomes. In the future, therapies that reduce fibrosis might help enhance the effectiveness of cancer treatments and improve patient survival.

This summary was initially drafted using artificial intelligence, then revised and fact-checked by the author.

## Introduction

The liver is the most common site of metastasis (occurring in over 50% of cases) and a leading cause of death among patients with colorectal cancer^1^. Although surgery remains the cornerstone of treatment for colorectal liver metastases (CRLM), the recurrence rates exceed 50% within the first 2 years following resection^2^, which is closely associated with poorer overall survival (OS). Furthermore, the efficacy of systemic treatments for unresectable CRLM is still suboptimal; for instance, the objective response rate for patients with CRLM treated with FOLFOX in combination with bevacizumab was approximately 50% (ref. ^3^). Consequently, a deeper understanding of the heterogeneity within the tumor microenvironment (TME) of liver metastasis (LM) may provide novel insights for therapeutic strategies targeting patients with CRLM^4^.

Tumor fibrosis was characterized by the pathological accumulation of extracellular matrix (ECM) components within tumor tissue^5^. Previous studies have demonstrated a correlation between tumor fibrosis and an immunosuppressive TME in various solid tumors, including breast cancer^6,7^, non-small-cell lung cancer^8^ and hepatocellular carcinoma^9^. This association may be attributed to the role of a rigid ECM as a physical barrier, as well as the phenotypic reprogramming of dendritic cells, macrophages and other immune cells, which leads to lymphocyte suppression^6–9^. Collectively, these factors may contribute to resistance to immunotherapy, particularly immune checkpoint inhibitors, ultimately resulting in adverse patient outcomes^10^. In recent years, antifibrotic therapies have increasingly shown promise in overcoming tumor resistance to immunotherapy and chemotherapy^11^. The influence of tumor fibrosis on the TME has become a focal point in clinical research.

However, in the context of CRLM, the clinical implications of tumor fibrosis in LM remain poorly understood. And a comprehensive characterization of the molecular features between tumor fibrosis present (Fibrosis+) LM and tumor fibrosis absent (Fibrosis−) LM at the single-cell resolution level remains lacking. This study aims to explore these issues from the aforementioned perspectives, providing clinicians with new perspectives for consideration.

## Methods

### Analysis of the association between tumor fibrosis in LM and survival

**Fig. 1:**
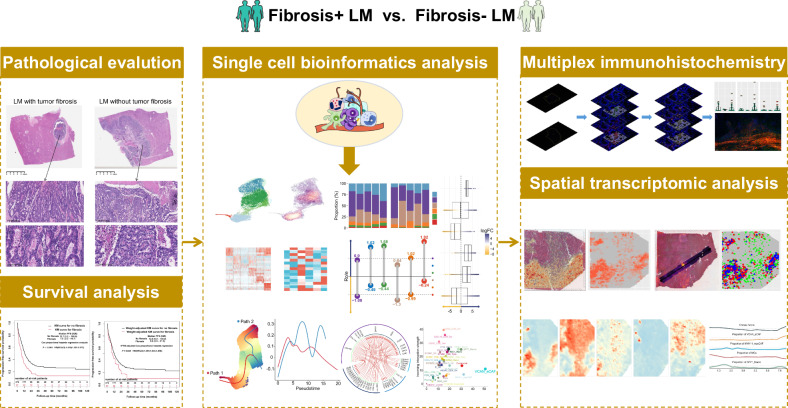
Workflow of this study. From left to right: Pathological assessment of liver metastasis fibrosis (top left), evaluation of clinical prognostic value (bottom left), overview of single-cell transcriptome analysis (center), multiplex immunofluorescence validation (top right), and spatial transcriptome analysis validation (bottom right).

A flow diagram illustrating the study’s analyses is depicted in Fig. 1. Ultimately, 471 patients with colorectal cancer LM receiving resection were retrospectively reviewed. Supplementary Table 1 details the clinicopathologic variables of interest for outcome analyses. Surgically resected specimens of LM were fixed with formalin, sectioned and stained with hematoxylin and eosin. Two certified pathologists independently evaluated tumor fibrosis and subsequently reached a consensus through collective assessment. In the case of a discordant outcome, the specimens were reviewed by a third independent expert pathologist.

Outcomes included progression-free survival (PFS) and OS. Multiple imputations via chained equations were performed to address the missing data. The observed variances in baseline characteristics were adjusted using the inverse probability of treatment weighting (IPTW) method. According to Rubin’s rules, estimated propensity scores from imputed datasets were amalgamated by computing the mean across all datasets. Standardized differences (SD), with a threshold of <0.1, were utilized to evaluate baseline characteristic balance. Adjusted Kaplan–Meier curves and log-rank tests based on IPTW were used to compare PFS and OS across groups. Furthermore, an inverse probability weighted Cox proportional hazards regression model was used to assess the relative hazard change (IPTW-adjusted hazards ratio (HR)). Statistical significance was set at *P* < 0.05 (two-sided), and all analyses were conducted using R version 4.1.1.

### Single-cell RNA sequencing for Fibrosis+ LM versus Fibrosis− LM

#### Sample collection and data processing for single-cell RNA sequencing

Single-cell RNA sequencing was performed on ten LM samples collected from ten patients with CRLM who underwent surgery without previous treatment. Clinical information for the enrolled patients is summarized in Supplementary Table 2. These samples were stratified into Fibrosis+ LM and Fibrosis− LM, with five samples in each group. The single-cell RNA sequencing data processing and analysis were performed as follows: the raw reads were computationally mapped to the GRCh38 human reference genome, with subsequent gene expression quantification at single-cell resolution performed using CellRanger software. These gene-cell count matrices were then imported into Seurat (v4.0.2) for downstream analysis. Following the quality control procedures detailed in the Supplementary Information, we implemented an established single-cell RNA sequencing analysis pipeline for clustering and annotation^12–14^. In brief, filtered cells underwent normalization and variance stabilization through SCTransform^15^, which simultaneously identified highly variable genes for downstream dimensional reduction. Principal component analysis was performed, with the optimal dimensionality determined via elbow plot assessment. Cell clustering was achieved through nearest-neighbor graph construction (FindNeighbors()) and community detection algorithms (FindClusters()), with results visualized using uniform manifold approximation and projection (UMAP) dimensionality reduction. For the cellular annotation, cluster-specific marker genes were identified using FindAllMarkers(), considering only significantly upregulated transcripts. The major cell types were classified on the basis of established markers^12–14^: epithelial cells (*EPCAM* and *KRT19*), T and natural killer (NK) cells (*CD3D*, *CD4*, *CD8A* and *PTPRC*), myeloid cells (*CD68*, *CD163*, *CD14* and *LYZ*), endothelial cells (*CLDN5* and *CDH5*), fibroblasts (*ACTA2*, *DCN* and *FAP*), mast cells (*TPSAB1*, *TPSB2* and *MS4A2*), B cells (*CD19*, *CD79A* and *MS4A1*) and plasma cells (*IGHG1*, *IGHA1* and *MZB1*). To resolve cellular heterogeneity, iterative subclustering was performed on major populations using the abovementioned analytical workflows (normalization, dimensional reduction and clustering), enabling the identification of distinct subtypes based on characteristic marker gene expression profiles. More detailed sample processing and quality control procedures can be found in the Supplementary Information.

#### Comparing cell type proportions

To evaluate differences in cellular composition within the TME between Fibrosis+ LM (*n* = 5) and Fibrosis− LM (*n* = 5) groups, we initially used the ratio of observed to expected cell numbers (Ro/e) (ref. ^16^) to assess the enrichment or depletion of individual cell clusters; an Ro/e value >1 indicates enrichment, whereas an Ro/e value <1 indicates depletion across the two groups. MiloR^17^, known for its discriminatory power in differential abundance, was also utilized to validate these findings. Subsequently, we used compared cell proportions of distinct cell subsets using the Wilcoxon rank-sum test and assessed the statistical significance.

#### Functional enrichment analysis

To characterize the functional heterogeneity of distinct cellular subpopulations, we performed gene set enrichment analysis using irGSEA to evaluate the well-established transcriptional signatures for T cell subsets (Supplementary Tables 3 and 4) and myeloid lineages (Supplementary Table 5), as previously defined in published studies^16,18,19^. This integrative analytical framework incorporates multiple single-cell enrichment algorithms—including AUCell, UCell, singscore, ssGSEA, JASMINE and Viper—to systematically profile the pathway activation dynamics across heterogeneous cell clusters. By implementing a consensus-based ranking strategy, we aggregated results from these complementary computational methods, thereby enabling a robust and multidimensional evaluation of pathway enrichment. In addition, aggregated normalized expression levels of predefined gene sets were computed using AddModuleScore function, enabling complementary insights into cellular phenotypes.

#### Pseudotime analysis

The R package Slingshot^20^ was then used to delineate potential lineage differentiation trajectories. All the trajectory starting points in this study were based on an integrated approach combining two methods: first, the automatic selection by the Slingshot algorithm and, second, biologically meaningful judgments supported by literature evidence. In cases of discrepancy, biological importance was prioritized. Moreover, generalized additive models were used to investigate the relationship between signature scores and pseudotimes derived from the Slingshot algorithm. Furthermore, the Geneswitches algorithm^21^ was applied to the pseudotime trajectories of specific cell clusters to predict pivotal changes in gene expression and pathway enrichment across pseudotime.

#### Tumor fibrosis responsiveness analysis by Augur

Augur^22^ represents a tailored machine learning approach designed to assess the priority of cell types within single-cell RNA sequencing datasets. By measuring the intensity of transcriptional responses across various cell types exposed to biological perturbations, Augur identifies the most responsive cell types under specific conditions.

#### Exploration of cell–cell interactions

To delineate the intercellular communication and differential signal pathways among distinct cell clusters within the TME, we utilized the Cellchat package^23^. In addition, the iTALK package^24^ was used to identify significant alterations in ligand–receptor (LR) interactions across cell clusters within the TME of the Fibrosis+ LM and Fibrosis− LM. The known LR pairs encompassing checkpoint, cytokine and growth factor interactions were investigated.

### Validation using mIHC/IF

To validate the differential distribution of cell clusters identified from single-cell RNA sequencing between Fibrosis+ LM and Fibrosis− LM, we used multiplex immunohistochemistry/immunofluorescence (mIHC/IF). The LM samples from patients with CRLM who underwent surgery without prior treatment were categorized into Fibrosis+ LM (*n* = 25) and Fibrosis− LM (*n* = 25) for the mIHC/IF analysis. The clinical details for these patients are provided in Supplementary Table 6. The detailed procedures for mIHC/IF can be found in the Supplementary Information. We conducted comparisons of specific cell subcluster densities between the Fibrosis+ LM and Fibrosis− LM.

### Validation using ST data

Spatial transcriptomics (ST) data analysis utilized two datasets (GSE225857 and GSE217414) obtained from the Gene Expression Omnibus. These datasets were processed using the Load10X_Spatial function from the Seurat package, focusing specifically on six LM samples from patients with CRLM (Supplementary Table 7). Tumor fibrosis levels in the ST data were evaluated using a curated gene signature as previously described^25^.

For deconvolution and cell type annotation, the integrated analysis of single-cell RNA sequencing and ST data was performed using the ‘CARD’ (v 1.1) package^26^ with default parameters. A ‘CARD’ object was initially created using the CreateCARDObject function, followed by applying CARD_deconvolution with default parameters to compute results. The relationship between cellular organization and tumor fibrosis was explored by evaluating the homotypic scores of selected cell clusters for spatial localization, following established methodologies^27^.

To further assess the correlation between tumor fibrosis levels and cell proportions across spatial locations, we utilized the SPATA2 R package^28^. The Seurat object was transformed into a Spata object using the transformSeuratToSpata function. Subsequently, notable variations in tumor fibrosis scores were visualized through a spatial trajectory created using the createTrajectories function. The changes in the cell proportions along this trajectory were visualized using the plotTrajectoryFeaturesDiscrete function. Moreover, the spatial colocalization of specific LR interactions was assessed using SpaGene^29^.

## Results

### Survival analyses for fibrosis in LM

Supplementary Fig. 1a, b illustrates the representative hematoxylin and eosin images of LM with (Supplementary Fig. 1a) and without (Supplementary Fig. 1b) tumor fibrosis. The clinicopathologic characteristics of included patients are summarized in Supplementary Table 1. The patients were stratified into two groups: fibrosis in LM (*n* = 97) and no fibrosis in LM (*n* = 374). The initial imbalances observed between the groups within the unadjusted cohort were effectively mitigated post adjustment (Supplementary Table 1).

The patients with fibrosis in LM demonstrated significantly worse PFS compared with those with no fibrosis in LM both before (median: 7.0 months versus 12.7 months, *P* < 0.001 in non-IPTW-adjusted Cox proportional hazards regression analysis, HR 1.610) and after adjustment (median: 9.9 months versus 12.4 months, *P* = 0.025 in IPTW-adjusted Cox proportional hazards regression analysis, HR 1.391) (Supplementary Fig. 2a, b). The patients with fibrosis in LM demonstrated significantly worse OS compared with those with no fibrosis in LM both before (median: 42.0 months versus 66.6 months, *P* = 0.003 in non-IPTW-adjusted Cox proportional hazards regression analysis, HR 1.678) and after adjustment (median: 46.0 months versus 58.8 months, *P* = 0.008 in IPTW-adjusted Cox proportional hazards regression analysis, HR 1.728) (Supplementary Fig. 2c, d).

### Single-cell RNA sequencing analysis of Fibrosis+ LM versus Fibrosis− LM

#### Global single-cell landscape for Fibrosis+ LM versus Fibrosis− LM

We performed single-cell RNA sequencing to compare the cellular composition between patients with high and Fibrosis− in CRLM. Across all samples, we identified eight main cell types, including T and NK cells (*CD3D*, *CD3G* and *PTPRC*), B cells (*CD19*, *CD79A* and *MS4A1*), plasma cells (*IGHG1*, *IGHA1* and *MZB1*), myeloid cells (*CD68*, *CD163*, *CD14* and *LYZ*), epithelial cells (*EPCAM* and *KRT19*), fibroblasts (*FAP*, *DCN* and *ACTA2*), endothelial cells (*CLDN5* and *CDH5*) and mast cells (*TPSAB1*, *TPSB2* and *MS4A2*)^12–14^ (Supplementary Fig. 3a, b). Notably, although Fibrosis− LM samples showed a tendency toward higher prevalence of T and NK cells and endothelial cells, and Fibrosis+ LM samples exhibited a relative enrichment of epithelial and myeloid cells (Supplementary Fig. 3c), these differences did not reach statistical significance (Supplementary Fig. 3d).

#### T cells reprogramming toward a suppressive microenvironment in TME of Fibrosis+ LM

**Fig. 2:**
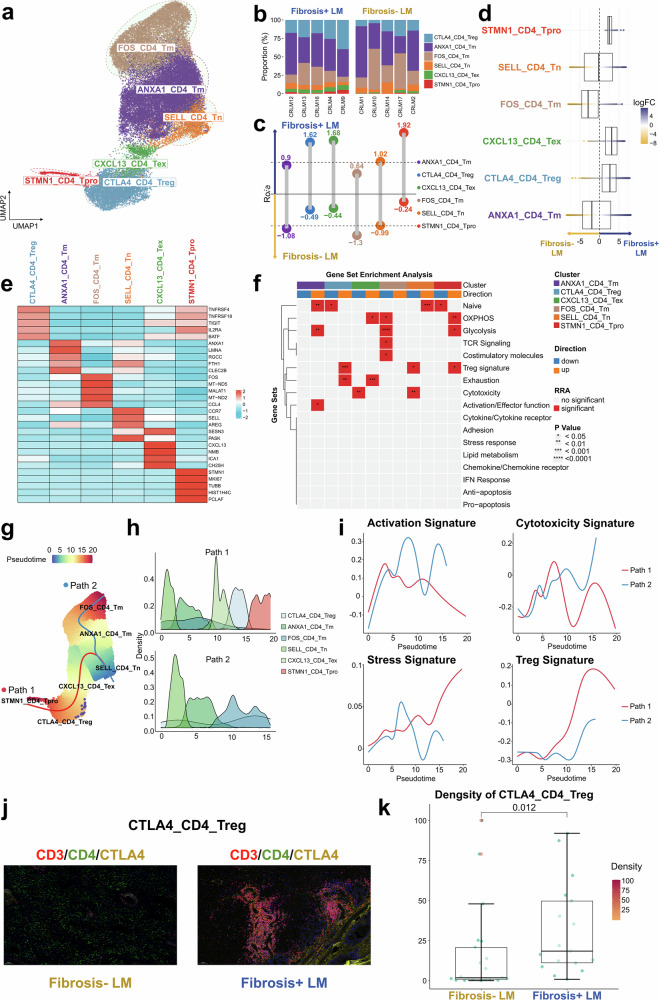
The landscape of *CD4*^+^ T cell. **a** A UMAP plot illustrating six *CD4*^+^ T cell clusters across all samples, color-coded by cell type. **b** The bar plot illustrates the relative proportion of *CD4*^+^ T cell clusters in each sample from the Fibrosis+ LM and Fibrosis− LM. **c** The lollipop chart displays the prevalence of different *CD4*^+^ T cell subgroups between the two groups, estimated by Ro/e. The subgroups favoring the Fibrosis+ LM are positioned above, whereas those favoring the Fibrosis− LM are positioned below. **d** The beeswarm and box plots depict the distribution of log_2_-fold differences in neighborhoods across various cell type clusters of *CD4*^+^ T cells, estimated by MiloR. The rightward positions indicate higher abundance in the Fibrosis+ LM, whereas the leftward positions indicate higher abundance in the Fibrosis− LM. **e** A heat map depicting the expression of marker genes across defined *CD4*^+^ T cell clusters. The color intensity reflects the average scaled gene expression. **f** A heat map via irGSEA visualizing the distribution of curated significant gene sets identified by robust rank aggregation across *CD4*^+^ T cell clusters. The number of asterisks in the grid’s upper half indicates the *P* value. **g** The Slingshot trajectory analysis of *CD4*^+^ T cell differentiation reveals two principal divergent trajectories. The cells are color-coded according to their pseudotime. **h** The density changes of *CD4*^+^ T cell subclusters along path 1 and path 2 are shown. **i** The two-dimensional plots showing expression scores for four representative gene signatures in cells of paths 1 and path 2, respectively, along the inferred pseudotime. **j** The mIHC/IF images showing the positive expression of CTLA4_CD4_T_reg_. **k** The quantification of the density of CTLA4_CD4_T_reg_ between Fibrosis+ LM and Fibrosis- LM group, Wilcox rank-sum test.

Tumor-infiltrating T cells play a pivotal role in the TME, yet their heterogeneous nature poses challenges for effective immunotherapy. Our analysis focused on *CD4*^+^ T cell clusters, delineated into *FOS*^+^*CD4*^+^ memory T cells (FOS_CD4_T_m_), *ANXA1*^+^*CD4*^+^ memory T cells (ANXA1_CD4_T_m_), *SELL*^+^*CD4*^+^ naive T cells (SELL_CD4_T_n_), *CXCL13*^+^*CD4*^+^ exhausted cells (CXCL13_CD4_T_ex_), *CTLA4*^+^*CD4*^+^ regulatory T cells (CTLA4_CD4_T_reg_) and *STMN1*^+^*CD4*^+^ proliferative T cells (STMN1_CD4_T_pro_) (Fig. 2a and Supplementary Table 8). Notable variations in *CD4*^+^ T cell states and compositions were observed between Fibrosis+ and Fibrosis− LMs (Fig. 2a, b). The analysis using Ro/e and MiloR algorithms revealed a higher proportion of CTLA4_CD4_T_reg_, CXCL13_CD4_T_ex_ and STMN1_CD4_T_pro_ cells in the Fibrosis+ LM, whereas ANXA1_CD4_T_m_ and FOS_CD4_T_m_ cells were more prevalent in the Fibrosis− LM (Fig. 2b–d). A statistical comparison of the cellular proportions confirmed significantly higher frequencies of CTLA4_CD4_T_reg_, CXCL13_CD4_T_ex_ and STMN1_CD4_T_pro_ cells in the Fibrosis+ LM group compared with the Fibrosis− LM group (*P* < 0.05) (Supplementary Fig. 4a).

Notably, CTLA4_CD4_T_reg_, CXCL13_CD4_T_ex_ and STMN1_CD4_T_pro_ cells exhibited the upregulation of exhaustion markers such as *TNFRSF4*, *TIGIT*, *BATF* and *IL2RA*, indicating impaired functionality^30^ (Fig. 2e). The STMN1_CD4_T_pro_ cells showed elevated levels of proliferative markers *STMN1* and *MKI67*, whereas CXCL13_CD4_T_ex_ cells demonstrated an increased expression of dysfunctional marker *CXCL13*, previously linked to immunotherapy responsiveness^31^. To evaluate the statistical significance of *CD4*^+^ T cell functional scores, we conducted comparative irGSEA analysis (Fig. 2f). STMN1_CD4_T_pro_ cells showed the significant upregulation of oxidative phosphorylation (OXPHOS), glycolysis and T_reg_ signature pathways, along with the downregulation of the naive pathway, consistent with their mature and proliferative phenotype. The SELL_CD4_T_n_ cells exhibited a significant upregulation of the naive pathway and downregulation of both T_reg_ signature and cytotoxicity pathways, matching their naive functional state. The CXCL13_CD4_T_ex_ cells displayed a marked upregulation of the exhaustion pathway and downregulation of cytotoxicity, aligning with their exhausted phenotype. The CTLA4_CD4_T_reg_ cells showed a significant upregulation of T_reg_ signature and exhaustion pathways, along with the downregulation of the naive pathway, corresponding to their mature immunosuppressive phenotype. The ANXA1_CD4_T_m_ cells demonstrated a significant upregulation of naive, activation and effector function and glycolysis pathways, consistent with their memory-like phenotype^12–14,16^.

Based on the aforementioned functional phenotypes, previous literature reports^14,32–34^ and the automatic selection by the Slingshot algorithm, SELL_CD4_T_n_ was selected as the most biologically plausible starting point for trajectory construction. The pseudotime analysis of *CD4*^+^ T cell subclusters revealed two predominant differentiation paths originating from SELL_CD4_T_n_ and progressing through ANXA1_CD4_T_m_. Path 1 progresses via CTLA4_CD4_T_reg_ to STMN1_CD4_T_pro_, whereas path 2 terminates at FOS_CD4_T_m_ (Fig. 2g, h). These distinct paths suggest divergent cellular fates, supported by dynamic gene expression patterns along the inferred pseudotime axis. Path 2 shows increased activation and cytotoxicity signatures, whereas path 1 exhibits enhanced stress response and T_reg_ signatures (Fig. 2i). Given the predominance of path 1 cells in the Fibrosis+ LM, *CD4*^+^ T cells in the TME may trend toward exhaustion or suppression, potentially impacting immunotherapy responsiveness. The elevated infiltration of CTLA4_CD4_T_reg_ cells, key immunosuppressive cells, in the Fibrosis+ LM was confirmed by mIHC/IF analysis (Fig. 2j, k and Supplementary Fig. 4b, c).

**Fig. 3:**
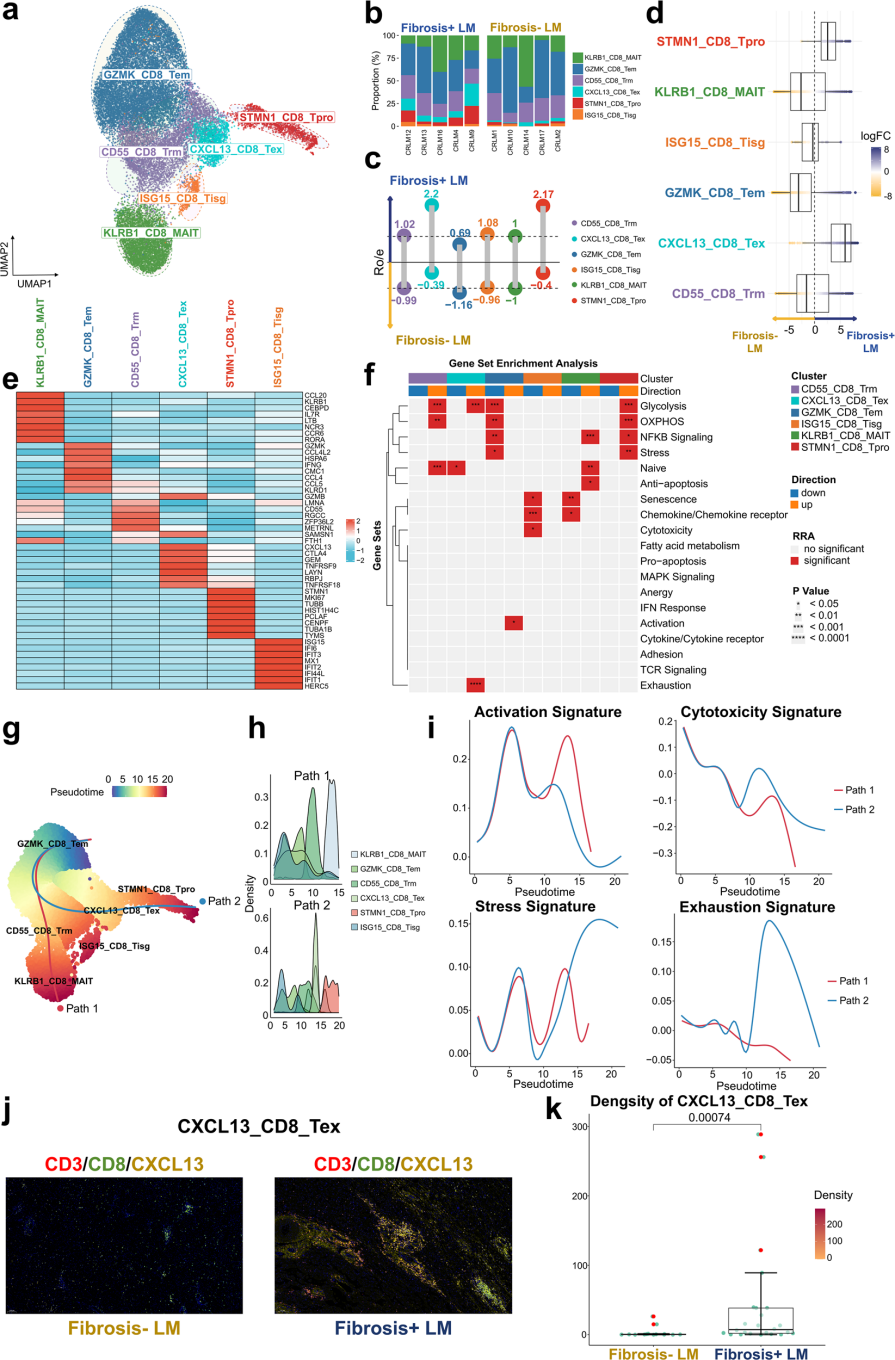
The landscape of *CD8*^+^ T cells. **a** A UMAP plot illustrating six *CD8*^+^ T cell clusters across all samples, color-coded by cell type. **b** The bar plot illustrates the relative proportion of *CD8*^+^ T cell clusters in each sample from the Fibrosis+ LM and Fibrosis− LM. **c** The lollipop chart displays the prevalence of different *CD8*^+^ T cell subgroups between the two groups, estimated by Ro/e. The subgroups favoring the Fibrosis+ LM are positioned above, whereas those favoring the Fibrosis− LM are positioned below. **d** The beeswarm and box plots depict the distribution of log_2_-fold differences in neighborhoods across various cell type clusters of *CD8*^+^ T cells, estimated by MiloR. The rightward positions indicate higher abundance in the Fibrosis+ LM, whereas the leftward positions indicate higher abundance in the Fibrosis− LM. **e** A heat map depicting the expression of marker genes across defined *CD8*^+^ T cell clusters. The color intensity reflects the average scaled gene expression. **f** A heat map via irGSEA visualizing the distribution of curated significant gene sets identified by robust rank aggregation across *CD8*^+^ T cells clusters. The number of asterisks in the grid’s upper half indicates the *P* value. **g** A Slingshot trajectory analysis of *CD8*^+^ T cell differentiation reveals two principal divergent trajectories. The cells are color-coded according to their pseudotime. **h** The density changes of *CD8*^+^ T cell subclusters along path 1 and path 2 are shown. **i** The two-dimensional plots showing the expression scores for four representative gene signatures in cells of paths 1 and path 2, respectively, along the inferred pseudotime. **j** The mIHC/IF images showing the positive expression of CXCL13_CD8_T_ex_. **k** The quantification of the density of CXCL13_CD8_T_ex_ between Fibrosis+ LM and Fibrosis− LM, Wilcox rank-sum test.

We further characterized *CD8*^+^ T cell subsets including *KLRB1*^+^*CD8*^+^ mucosal-associated invariant T cells (KLRB1_CD8_MAIT), *CD55*^+^*CD8*^+^ resident-memory T cells (CD55_CD8_T_rm_), *GZMK*^+^*CD8*^+^ effective-memory T cells (GZMK_CD8_T_em_), *ISG15*^+^*CD8*^+^ interferon response T cells (ISG15_CD8_T_isg_), *CXCL13*^+^*CD8*^+^ exhausted T cells (CXCL13_CD8_T_ex_) and *STMN1*^+^*CD8*^+^ proliferative T cells (STMN1_CD8_T_pro_) (Fig. 3a and Supplementary Table 9). Ro/e and MiloR analysis revealed a higher proportion of CXCL13_CD8_T_ex_ and STMN1_CD8_T_pro_ cells in the Fibrosis+ LM, whereas GZMK_CD8_T_em_ cells were more prevalent in the Fibrosis− LM (Fig. 3b–d). The statistical comparison of cellular proportions confirmed significantly higher frequencies of CXCL13_CD8_T_ex_ and STMN1_CD8_T_pro_ in the Fibrosis+ LM group compared with the Fibrosis− LM group (*P* < 0.05) (Supplementary Fig. 5a).

The CXCL13_CD8_T_ex_ cells exhibited upregulated exhaustion markers (*TNFRSF9* and *CTLA4*) and *CXCL13*, indicative of impaired functionality (Fig. 3e). By contrast, STMN1_CD8_T_pro_ cells displayed elevated levels of proliferative markers (*STMN1* and *MKI67*), and GZMK_CD8_T_em_ cells showed increased cytotoxicity markers (*GZMK* and *KLRD1*) and activation markers (*IFNG*). Consistent with the gene expression patterns, we performed irGSEA comparative analysis to evaluate the statistical significance of *CD8*^+^ T cell functional profiles (Fig. 3f). The STMN1_CD8_T_pro_ cells showed a significant upregulation of glycolysis, OXPHOS, NF-κB signaling and stress pathways, consistent with their mature and proliferative phenotype. The KLRB1_CD8_MAIT cells exhibited a significant upregulation of naive, NF-κB signaling and anti-apoptosis pathways, along with the downregulation of both senescence and chemokine receptor pathways. The GZMK_CD8_T_em_ cells displayed a marked upregulation of activation pathways but a downregulation of glycolysis, OXPHOS, NF-κB signaling and stress response pathways, suggesting their potential positioning at the opposite developmental end from STMN1_CD8_T_pro_, CXCL13_CD8_T_ex_ showed a significant upregulation of glycolysis and exhaustion pathways but a downregulation of the naive pathway, corresponding to their mature immunosuppressive phenotype. CD55_CD8_T_rm_ demonstrated a significant upregulation of naive, glycolysis and OXPHOS pathways, consistent with their resident memory-like phenotype.

On the basis of the comprehensive functional characterization, supporting evidence from previous studies^14,32–34^ and the automatic selection by the Slingshot algorithm, GZMK_CD8_T_em_ was identified as the most biologically appropriate origin for trajectory reconstruction. The trajectory analysis identified two primary paths for *CD8*^+^ T cells originating from GZMK_CD8_T_em_ and progressing through CD55_CD8_T_rm_. Path 1 terminated in KLRB1_CD8_MAIT, whereas path 2 continued through CXCL13_CD8_T_ex_ to STMN1_CD8_T_pro_ (Fig. 3g, h). The dynamic gene signature expression indicated that Path 2 exhibited heightened stress response and exhaustion signatures, although both paths showed decreased cytotoxicity signatures (Fig. 3i). Given the predominance of path 2 cells in the Fibrosis+ LM, *CD8*^+^ T cells in this TME context may be predisposed to exhaustion. Validation through mIHC/IF analysis confirmed the increased infiltration of the primary exhausted T cell subset, CXCL13_CD8_T_ex_, in the Fibrosis+ LM (Fig. 3j, k and Supplementary Fig. 5b, c). In summary, the TME of the Fibrosis+ LM exhibits a distinct immunosuppressive T cell landscape characterized by enriched T_reg_s and exhausted *CD8*^+^ T cells, potentially leading to enhanced immune suppression and exhaustion.

#### Macrophages displaying profibrotic phenotye in the TME of Fibrosis+ LM

**Fig. 4:**
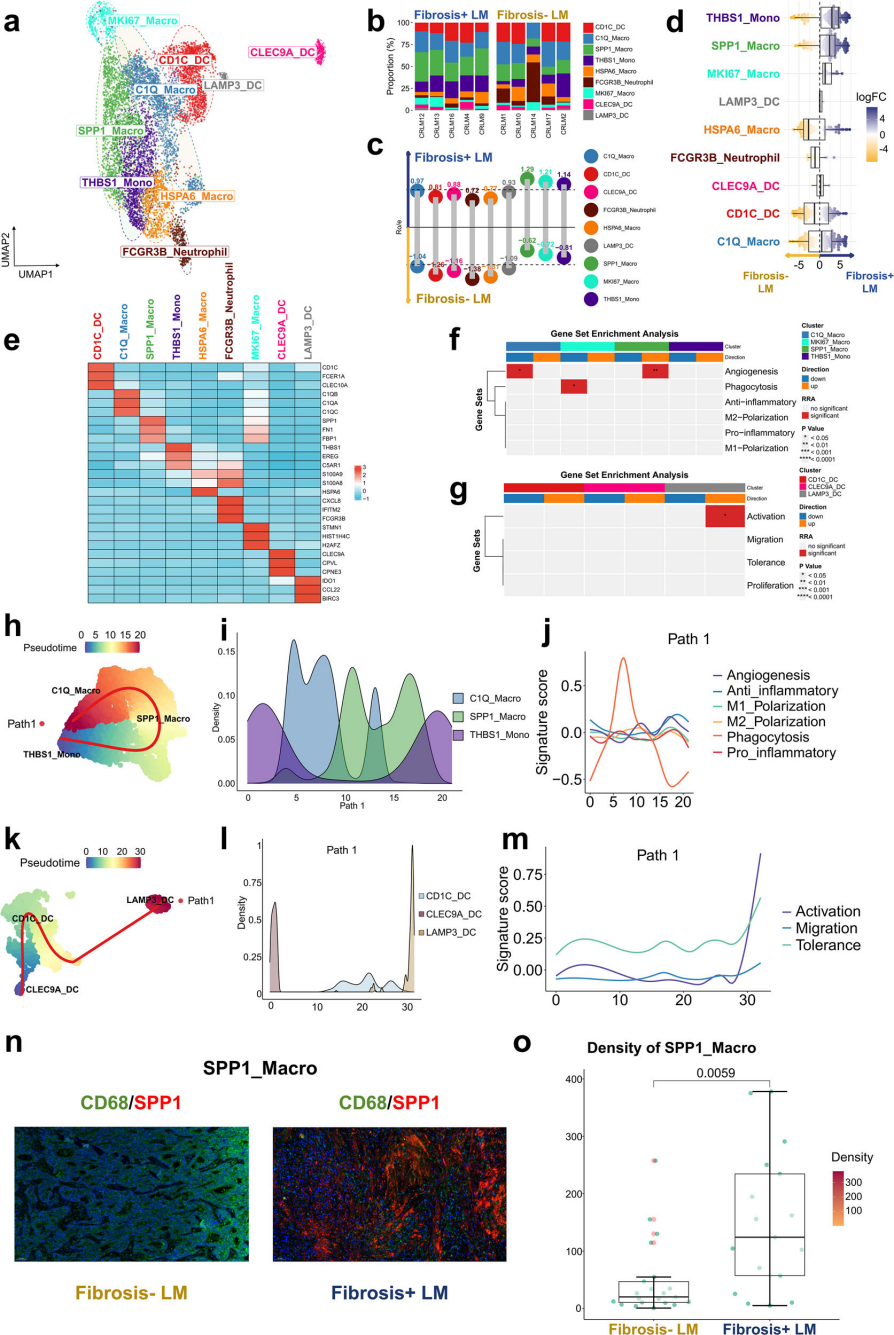
The landscape of myeloid cells. **a** A UMAP plot illustrating nine myeloid cell clusters across all samples, color-coded by cell type. **b** The bar plot illustrates the relative proportion of myeloid cell clusters in each sample from the Fibrosis+ LM and Fibrosis− LM. **c** The lollipop chart displays the prevalence of different myeloid cell subgroups between the two groups, estimated by Ro/e. The subgroups favoring the Fibrosis+ LM are positioned above, whereas those favoring the Fibrosis− LM are positioned below. **d** The beeswarm and box plots depict the distribution of log_2_-fold differences in neighborhoods across various cell type clusters of myeloid cell, estimated by MiloR. The rightward positions indicate higher abundance in the Fibrosis+ LM, whereas the leftward positions indicate higher abundance in the Fibrosis− LM. **e** A heat map depicting the expression of marker genes across defined myeloid cell clusters. The color intensity reflects the average scaled gene expression. **f** A heat map via irGSEA visualizing the distribution of curated significant gene sets identified by robust rank aggregation across macrophage as well as monocyte cells clusters. The number of asterisks in the grid’s upper half indicates the *P* value. **g** A heat map visualizing the distribution of curated significant gene sets identified by robust rank aggregation across DC cell clusters. The number of asterisks in the grid’s upper half indicates the *P* value. **h** A Slingshot trajectory analysis of macrophage as well as monocyte differentiation reveals one principal divergent trajectory. The cells are color-coded according to their pseudotime. **i** The density changes of macrophage subclusters along path 1 are shown. **j** The two-dimensional plots showing expression scores for seven representative gene signatures in cells of paths 1, along the inferred pseudotime. **k** A Slingshot trajectory analysis of DC differentiation reveals one principal divergent trajectories. The cells are color-coded according to their pseudotime. **l** The density changes of DC subclusters along path 1 are shown. **m** The two-dimensional plots showing expression scores for three representative gene signatures in cells of paths 1 along the inferred pseudotime. **n** The mIHC/IF images showing the positive expression of SPP1_Macro. **o** The quantification of the density of SPP1_Macro between Fibrosis+ LM and Fibrosis− LM group, Wilcox rank-sum test.

To investigate myeloid populations in CRLM, myeloid cells were categorized into subsets including *MKI67*^+^ macrophages (MKI67_Macro), *SPP1*^+^ macrophages (SPP1_Macro), *THBS1*^+^ monocytes (THBS1_Mono), *HSPA6*^+^ macrophages (HSPA6_Macro), *C1Q*^+^ macrophages (C1Q_Macro), *C1DC*^+^ dendritic cells (C1DC_DC), *LAMP3*^+^ dendritic cells (LAMP3_DC), *CLEC9A*^+^ dendritic cells (CLEC9A_DC) and *FCGR3B*^+^ neutrophils (FCGR3B_Neutrophil) (Fig. 4a and Supplementary Table 10). The Ro/e and MiloR analyses showed higher proportions of THBS1_Mono, SPP1_Macro and MKI67_Macro in the Fibrosis+ LM, whereas HSPA6_Macro, CD1C_DC and FCGR3B_Neutrophil were more prevalent in the Fibrosis− LM (Fig. 4b–d). The statistical analysis revealed a significantly higher proportion of SPP1_Macro in the Fibrosis+ LM group compared with the Fibrosis− LM group (Supplementary Fig. 6a).

SPP1_Macro exhibited an elevated expression of profibrotic markers *SPP1*, *FN1* and *FBP1*, previously associated with fibrotic macrophages^29^. C1Q_Macro showed an increased expression of complement system genes such as *C1QA*, *C1QB* and *C1QC*. The MKI67_Macro population exhibited upregulated proliferation markers *STMN1* and *MKI67* while displaying mixed characteristics of both SPP1_Macro and C1Q_Macro (Fig. 4e). This cluster was probably grouped owing to its highly expressed cell cycle-related genes and was therefore excluded from downstream trajectory analysis to avoid interference with the differentiation path inference. The HSPA6_Macro populations exhibited a high expression of heat-shock-protein-encoding genes, probably associated with the aforementioned tissue dissociation procedure^35–37^, and were consequently excluded from subsequent analyses. FCGR3B_Neutrophil demonstrated an enhanced expression of neutrophil maturation markers, including *C5AR1* as well as neutrophil markers *S100A8* and *S100A9*^37–39^ (Fig. 4e). It is worth noting that because neutrophils typically contain low RNA levels, we further validated this cell population’s identity using multiple approaches. First, we compared the expression of established neutrophil markers (*CXCR2*, *FCGR3B* and *CSF3R*)^37–39^, which showed significantly higher expression in this subset than in other cell populations (Supplementary Fig. 7a–c). In addition, we assessed the signature scores of neutrophil using gene sets from three previous studies^40–42^ (Supplementary Table 11) via the AddModuleScore, which similarly demonstrated significantly higher scores in FCGR3B_Neutrophil compared with others (Supplementary Fig. 7d–f). Together, these consistent findings robustly support our initial annotation. To assess the functional enrichment significance in macrophages and monocytes, we conducted comparative analysis using irGSEA (Fig. 4f, g). SPP1_Macrophage showed a significant upregulation of angiogenesis pathways (Fig. 4f), consistent with their established proangiogenic function^43^. Similarly, LAMP3_DC exhibited a pronounced enrichment of immune activation pathways (Fig. 4g), matching their reported mature regulatory phenotype^37–42^.

According to previous studies^13,44,45^ and the automatic selection by the Slingshot algorithm, THBS1_Mono was selected as the progenitor population for developmental trajectory analysis. Pseudotemporal reconstruction revealed a predominant differentiation pathway, with macrophages and monocytes deriving from THBS1_Mono, transitioning through SPP1_Macro and ultimately maturing into C1Q_Macro (Fig. 4h, i). Notably, phagocytic activity scores exhibited a transient peak followed by a rapid decline along this developmental trajectory (Fig. 4j). Consistent with previous reports^18,44,46^ and the automatic selection by the Slingshot algorithm, LAMP3_DC was identified as the terminal population in the developmental trajectory analysis. The DC differentiation trajectories revealed a single path starting at CLEC9A_DC, passing through CD1C_DC and culminating at LAMP3_DC (Fig. 4k, l), with increased activation, migration and tolerance signatures along this trajectory (Fig. 4m).

The independent verification through mIHC/IF analysis confirmed the enhanced infiltration of the profibrotic macrophage subset, SPP1_Macro, in the Fibrosis+ LM (Fig. 4n, o and Supplementary Fig. 6b, c). Overall, the TME of the Fibrosis+ LM was characterized by the presence of profibrotic macrophages, reduced complement activation in macrophages and decreased numbers of antigen-presenting DCs.

#### TME of Fibrosis+ LM characterized by the presence of profibrotic and ECM-remodeling fibroblasts

**Fig. 5:**
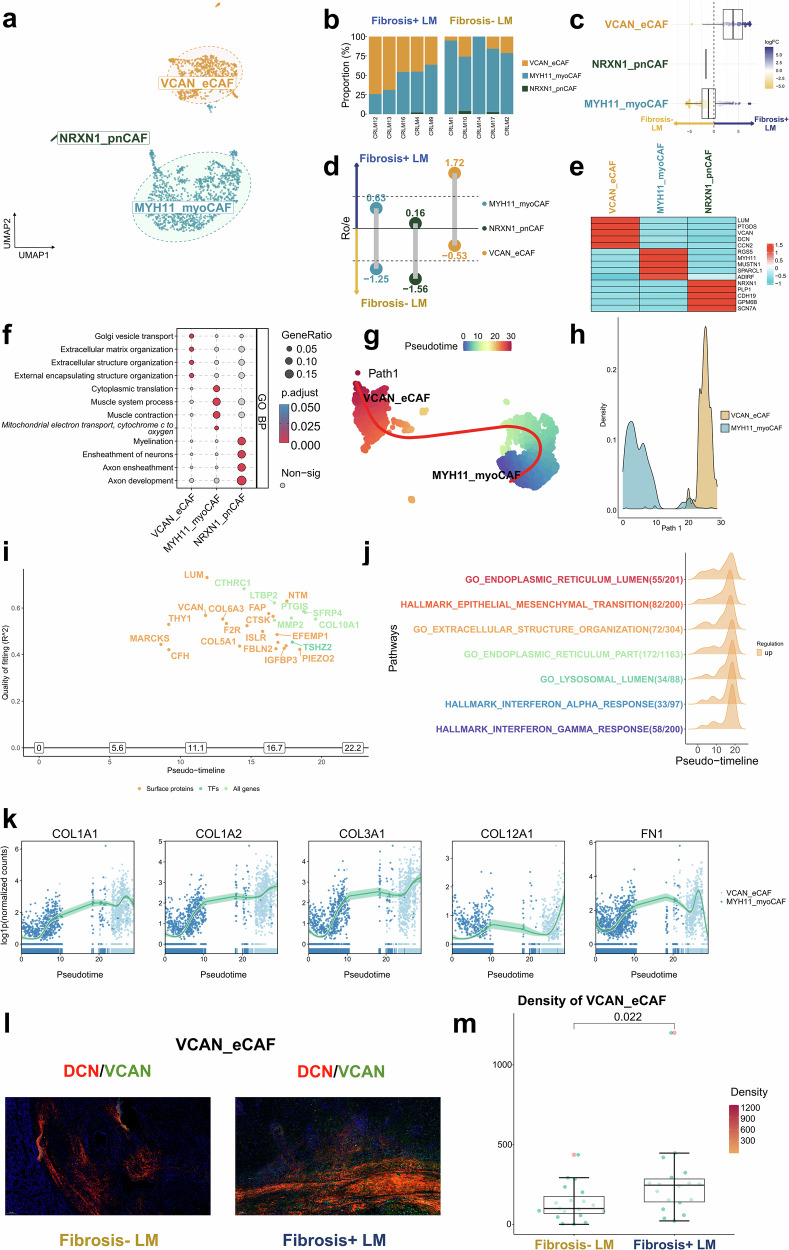
The landscape of CAFs. **a** A UMAP plot illustrating three CAF clusters across all samples, color-coded by cell type. **b** The bar plot illustrates the relative proportion of CAF clusters in each sample from the Fibrosis+ LM and Fibrosis− LM. **c** The lollipop chart displays the prevalence of different CAF subgroups between the two groups, estimated by Ro/e. The subgroups favoring the Fibrosis+ LM are positioned above, whereas those favoring the Fibrosis− LM are positioned below. **d** The beeswarm and box plots depict the distribution of log_2_-fold differences in neighborhoods across various cell type clusters of CAF, estimated by MiloR. The rightward positions indicate higher abundance in the Fibrosis+ LM, whereas the leftward positions indicate higher abundance in the Fibrosis− LM. **e** A heat map depicting the expression of marker genes across defined CAF clusters. The color intensity reflects the average scaled gene expression. **f** A heat map showing the functional pathways activated in different CAF subclusters using GO analysis. The heat map is based on scaled gene signature scores. **g** A Slingshot trajectory analysis of CAF differentiation reveals one principal divergent trajectories. The cells are color-coded according to their pseudotime. **h** The density changes of CAF subclusters along path 1 are shown. **i** The pivotal upregulated genes and surface proteins orchestrating this regulatory process along the inferred pseudotime, estimated by GeneSwitches. **j** The biological processes triggered to orchestrate this regulatory process along the inferred pseudotime, estimated by GeneSwitches. **k** The changes of common matrix genes along path 1 are shown. **l** The mIHC/IF images showing the positive expression of VCAN_eCAF. **m** The quantification of the density of VCAN_eCAF between the Fibrosis+ LM and Fibrosis− LM groups, Wilcox rank-sum test.

In this study, fibroblasts were classified into three subsets: versican (*VCAN*)^+^ matrix fibroblasts (VCAN_eCAF), *MYH11*^+^ myofibroblasts (MYH11_myoCAF) and *NRXN1*^+^ peripheral nerve-like fibroblasts (NRXN1_pnCAF) (Fig. 5a and Supplementary Table 12). An analysis using Ro/e and MiloR indicated that the Fibrosis+ LM exhibited higher proportions of VCAN_eCAF, whereas the Fibrosis− LM showed increased levels of MYH11_myoCAF (Fig. 5b–d). The statistical analysis revealed distinct differences in cancer-associated fibroblast (CAF) subtype distribution between fibrosis groups: VCAN_eCAF were significantly enriched in Fibrosis+ LM samples, whereas MYH11_myoCAF predominated in Fibrosis− LM samples (*P* < 0.05; Supplementary Fig. 8a).

Using the AddModuleScore to evaluate signature scores of different CAF subtypes from previous studies (following Liu et al.’s method^47^, see Supplementary Table 13), we performed a classification of the three identified CAF populations in this study. Notably, VCAN_eCAF exhibited the highest ‘Liver1_mCAF’ signature scores, consistent with the established matrix-producing CAF phenotype^48^ (Supplementary Fig. 8b). This subtype notably expresses elevated levels of genes encoding ECM proteins such as *LUM*, *VCAN* and *DCN*, highlighting its strong ECM protein secretion capability (Fig. 5e). To more accurately characterize the functional properties of fibroblasts, we performed a gene ontology (GO) analysis using limma v3.52.4 to evaluate pathway activities^14^, with an adjusted *P* value threshold of <0.05 considered statistically significant for identifying enriched pathways (Fig. 5f). The results further confirmed that VCAN_eCAF is significantly enriched in pathways related to ECM remodeling, including ‘ECM organization’ and ‘extracellular structure organization’. MYH11_myoCAF exhibited the highest scores for the ‘Pan-cancer1_CAF_myo(C1)’ signature, suggesting a myofibroblast phenotype^49^, consistent with its high expression of *MYH11* and *MUSTN1* (Fig. 5e and Supplementary Fig. 8b). The functional enrichment analysis revealed that this subtype was significantly enriched in pathways related to muscle contraction and tissue repair (Fig. 5f). Although NRXN1_pnCAF represented a minor population, our study identified that it exhibited the highest ‘Pan-cancer1_CAF_PN(C7)’ scores, corresponding to peripheral nerve-associated CAFs (Supplementary Fig. 8b) as previously reported^49^. The GO analysis (Fig. 5f) demonstrated that NRXN1_pnCAF is enriched in pathways such as the ‘ensheathment of neurons’, consistent with its gene expression profile, further supporting its potential neurorelated characteristics.

Given the rarity and low abundance of NRXN1_CAF, we excluded this population from subsequent trajectory analysis to avoid potential interference with the differentiation inference. On the basis of previous studies^47,50,51^ and the automatic selection by the Slingshot algorithm, MYH11_myoCAF was selected as the progenitor population for developmental trajectory analysis. The developmental trajectory originated from MYH11_myoCAF and culminated in VCAN_eCAF, indicating that VCAN_eCAF represents an activated state (Fig. 5g, h). The GeneSwitches analysis identified key upregulated genes during the transition from MYH11_myoCAF to VCAN_eCAF, predominantly involving collagen family members (*COL5A1*, *COL6A3* and *COL10A1*) and ECM regulators (*VCAN*, *LUM*, *CTHRC1*, *EFEMP1*, *LTBP2* and *MMP2*)^52^, which orchestrated ECM reorganization (GO_EXTRACELLULAR_STRUCTURE_ORGANIZATION) and EMT (HALLMARK_EPITHELIAL_MESENCHYMAL_TRANSITION) through fibrillar collagen assembly^53,54^. The transition was further characterized by endoplasmic reticulum lumen expansion (GO_ENDOPLASMIC_RETICULUM_LUMEN), marked by *IGFBP3*, *EFEMP1* and *SFRP4*, reflecting heightened protein processing to meet secretory demands^55,56^, along with lysosomal lumen activity (GO_LYSOSOMAL_LUMEN) driven by *CTSK* and *MMP2*, indicative of ECM degradation during remodeling^57,58^. Moreover, the inflammatory responses (HALLMARK_INTERFERON_ALPHA/GAMMA_RESPONSE) were integrated through interferon-modulating genes (*CFH*, *ISLR* and *THY1*), linking immune signaling with stromal reprogramming^59,60^. Collectively, these molecular features delineate the functional specialization of VCAN_eCAF in coordinating ECM remodeling, secretory adaptation and immune-stromal crosstalk (Fig. 5i, j). Key matrix genes such as *COL1A1*, *COL1A2*, *COL3A1*, *COL12A1* and *FN1*^61^ were found to be upregulated during the transition from MYH11_myoCAF to VCAN_eCAF (Fig. 5k).

Moreover, through mIHC/IF analysis, we independently confirmed the increased infiltration of VCAN_eCAF in the Fibrosis+ LM (Fig. 5l, m and Supplementary Fig. 8c, d). In conclusion, the TME of the Fibrosis+ LM is characterized by the presence of fibroblasts actively involved in profibrotic processes and ECM remodeling, underscoring their substantial role in shaping the TME in CRLM with Fibrosis+.

#### Malignant epithelial cells with a more activated and invasive phenotype identified in TME of Fibrosis+ LM

**Fig. 6:**
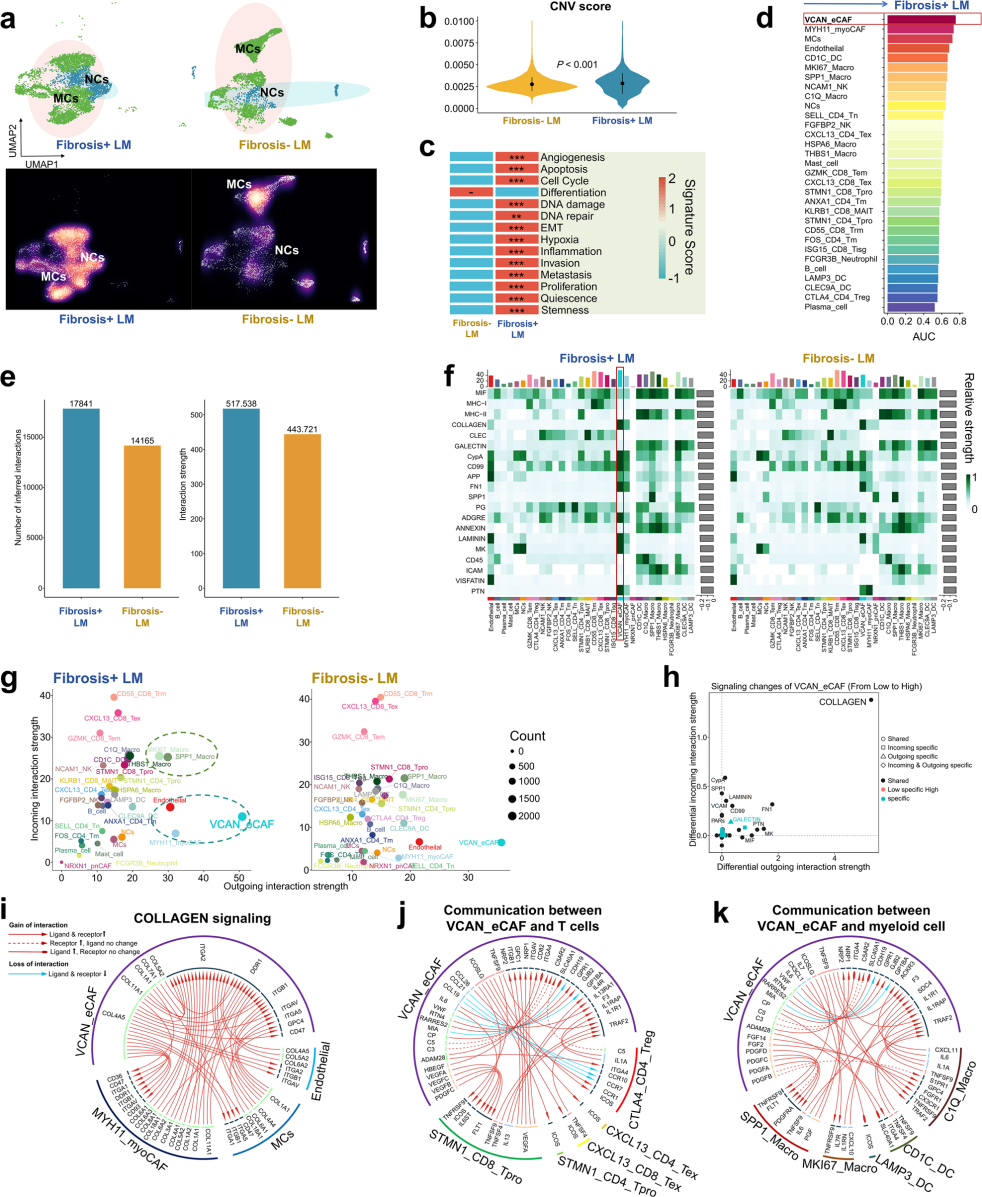
The landscape of epithelial cells and the cell–cell communication analysis. **a** A UMAP view of MCs and NCs (top) and cell density (bottom) demonstrating the distribution between Fibrosis+ LM and Fibrosis− LM. High relative cell density is shown as bright magma. **b** The violin plot compared the CNV scores of MCs between Fibrosis+ LM and Fibrosis− LM, Wilcox rank-sum test. **c** A heat map illustrating the expression profiles of curated gene signatures in MCs between the Fibrosis+ LM and Fibrosis− LM. The heat map is based on scaled gene signature scores, Wilcox rank-sum test, *P* value: **≤0.01, ***≤0.001. **d** A bar chart displaying the Augur scores of cell types across all cell clusters. The length of each bar indicates the Augur score, with longer bars indicating a stronger association with the Fibrosis+ phenotype. **e** Cellchat compares the total number of interactions and interaction strength of the inferred cell–cell communication networks between Fibrosis+ LM and Fibrosis− LM. **f** A heat map showing the contribution of signals (signaling pathways or LR pairs) to cell groups in terms of the overall signaling between Fibrosis+ LM and Fibrosis− LM, estimated by Cellchat. **g** The two-dimensional plots showing the incoming and outgoing interaction strengths for each of the cell types between Fibrosis+ LM and Fibrosis− LM. **h** The signaling changes of VCAN_eCAF from Fibrosis− LM to Fibrosis+ LM, estimated by Cellchat. **i** The significantly upregulated or downregulated LR pairs involved in ‘COLLAGEN’ signaling between VCAN_eCAF and other cells, such as MYH11_myoCAF, endothelial cells and MCs, estimated by iTALK. **j**, **k** The significantly upregulated or downregulated LR pairs in all signaling pathways between VCAN_eCAF and other cells, including T cells (**j**) and myeloid cells (**k**), estimated by iTALK.

Copy number variation (CNV) analysis was performed across all epithelial cell populations, utilizing 500 T or NK cells, 500 plasma cells and 500 B cells as reference controls (Supplementary Fig. 9a). Compared with reference cells, notable CNV aberrations were detected in the majority of epithelial cells (Supplementary Fig. 9b, c). The subsequent classification identified two distinct epithelial subsets: malignant cells (MCs) and normal epithelial cells (NCs) (Fig. 6a). Based on the biological characteristics of our samples (derived from LM lesions), the observed NCs were restricted to two potential origins: hepatocytes or cholangiocytes. To clarify their identity, we used two approaches: examining the expression of hepatocyte markers (*ALB*, *AFP* and *ARG1*) and cholangiocyte markers (*KRT8*, *KRT19* and *SOX9*)^62–64^ in NCs, and evaluating gene signature scores for hepatocytes and cholangiocytes using AddModuleScore with reference to a published single-cell RNA sequencing dataset of healthy liver tissue^62^ (Supplementary Table 14). The analysis revealed that these cells highly expressed cholangiocyte markers but showed negligible expression of hepatocyte markers (Supplementary Fig. 9d). Furthermore, NCs exhibited the highest scores for the ‘cholangiocyte’ signature, indicating a cholangiocyte-like profile (Supplementary Fig. 9e). Collectively, these findings suggested that the NCs were more likely to be of cholangiocyte origin. Notably, MCs in Fibrosis+ LM exhibited significantly elevated CNV scores compared with Fibrosis− LM (Fig. 6b). Comparative analysis of classical hallmarks of tumor biological signatures revealed that Fibrosis+ LM-derived MCs exhibited markedly higher scores for these traits (Fig. 6c). Cell cycle phase distribution analysis uncovered a progressive increase in the proportion of proliferating MCs (S and G2/M phases) within Fibrosis+ LM, indicative of heightened proliferative activity (Supplementary Fig. 10a). The CytoTRACE-based evaluation further identified significantly elevated cancer stemness properties in Fibrosis+ LM-derived MCs relative to Fibrosis− LM counterparts (Supplementary Fig. 10b). The pathway activity profiling via PROGENy revealed distinct oncogenic pathway activation patterns: Wnt, NF-κB and TNFα signaling pathways were predominantly enriched in Fibrosis+ LM-derived MCs, whereas Estrogen and Trail pathways showed preferential enrichment in Fibrosis− LM-derived MCs (Supplementary Fig. 10c).

The pseudotime trajectory reconstruction delineated developmental paths from NCs to Fibrosis+ LM-derived MCs versus Fibrosis− LM-derived MCs (Supplementary Fig. 10d). The transcriptomic profiling along these trajectories demonstrated the notable upregulation of Wnt pathway-related genes specifically in Fibrosis+ LM-derived MCs (Supplementary Fig. 10e). Collectively, these findings indicate that Fibrosis+ LM-derived MCs exhibit distinct molecular characteristics from Fibrosis− LM-derived MCs, displaying enhanced malignant potential potentially driven by the Wnt pathway activation. The therapeutic targeting of this pathway may represent a strategic approach for Fibrosis+ LM management.

#### VCAN_eCAF playing a potential role in reshaping the TME of Fibrosis+ LM

Using the Augur algorithm, VCAN_eCAF was identified as one of the most transcriptionally responsive cell types to the Fibrosis+ (Fig. 6d), followed by MYH11_myoCAF and MCs. A comparative analysis of the key fibroblast subpopulations revealed distinct characteristics and functional states. MYH11_myoCAF, enriched in nonfibrotic regions (Fibrosis− LM) and expressing smooth muscle-associated genes (*MYH11* and *MUSTN1*), may represent a progenitor and resident state primarily associated with structural and contractile functions. By contrast, VCAN_eCAF, substantially expanded in fibrotic regions (Fibrosis+ LM) and highly expressing core ECM genes (*LUM*, *VCAN* and collagens), may constitute an activated, terminal state that dominates the pathological ECM remodeling (Fig. 5a, f, k). Critically, the developmental trajectory analysis demonstrated that MYH11_myoCAF serve as the precursor population differentiating into VCAN_eCAF under profibrotic stimuli (Fig. 5g, h). Given the dominant enrichment of VCAN_eCAF in fibrotic lesions and their direct role as the primary effector cells executing pathological ECM deposition, subsequent analyses focus on elucidating the mechanisms by which this subpopulation drives fibrosis. To deepen our understanding of VCAN_eCAF’s role within the TME of the Fibrosis+ LM, we examined the complex network of intercellular communication. Compared with the Fibrosis− LM, there was a notable increase in both the frequency and intensity of interactions among various cellular subpopulations within the TME of the Fibrosis+ LM (Fig. 6e). VCAN_eCAF emerged as a primary source of signals within the broader signaling landscape (Fig. 6f). A comparative analysis with the Fibrosis− LM highlighted significant alterations in the strengths of both incoming and outgoing interactions, particularly affecting VCAN_eCAF (Fig. 6g). Moreover, the roles of endothelial cells, MYH11_myoCAF, SPP1_Macro and MKI67_Macro became more pronounced, indicating a more intricate network of cellular interactions within the TME of the Fibrosis+ LM (Fig. 6g). These observations, supported by our comprehensive analysis of transcriptional responsiveness (Fig. 6d), cell–cell communication networks (Fig. 6e–g) and pathway activation patterns (Fig. 5i, j), collectively demonstrate the potential involvement of VCAN_eCAF in shaping the TME dynamics of this cohort through its dual role as both a primary signal source and a key responder to fibrotic stimuli.

The further exploration of signaling variations in VCAN_eCAF between the low and Fibrosis+ LMs revealed a notable relevance of ‘COLLAGEN’, suggesting that VCAN_eCAF may influence the TME through the activation of collagen signaling pathways (Fig. 6h). Utilizing the iTALK algorithm, we identified specific LR pair alterations, highlighting enhanced interactions such as *COL1A1*–*ITGB1*, *COL4A5*–*ITGA2* and *COL5A2*–*DDR1* between VCAN_eCAF and other cell types including MYH11_myoCAF, endothelial cells and MCs within the Fibrosis+ LM (Fig. 6i). In addition, augmented LR partnerships such as *ICOSLG*–*ICOS* and *TNFSF9*–*TRAF2* from VCAN_eCAF to various cell types including STMN1_CD8_T_pro_, STMN1_CD4_T_pro_, CXCL13_CD4_T_ex_, CXCL13_CD8_T_ex_, CTLA4_CD4_T_reg_, SPP1_Macro, MKI67_Macro, C1Q_Macro, CD1C_DC and LAMP3_DC were observed (Fig. 6j, k). These findings suggest that VCAN_eCAF may contribute to the progression of tumor fibrosis and regulate the infiltration of T cells and myeloid cells within the TME of the Fibrosis+ LM through these intricate LR interactions.

### ST data displaying landscape of VCAN_eCAF in LM

**Fig. 7:**
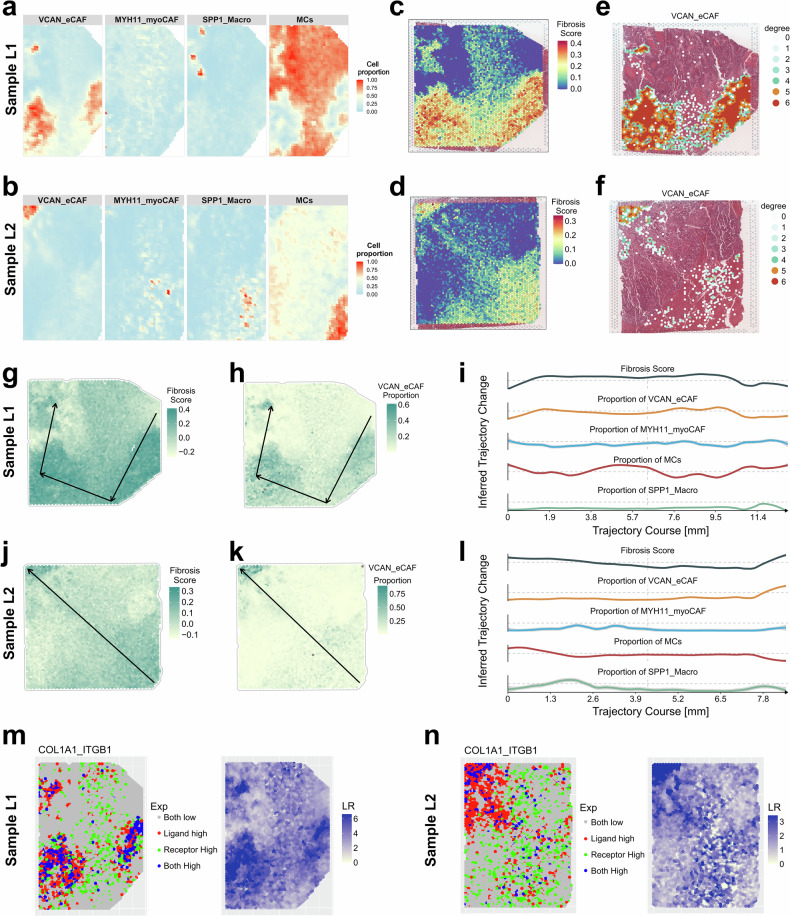
The relationship between VCAN_eCAF and tumor fibrosis was assessed using spatial RNA sequencing. **a** The spatial distribution of VCAN_eCAF, MYH11_myoCAF, SPP1_Macro and MCs proportions in sample L1, estimated by CARD. **b** The spatial distribution of VCAN_eCAF, MYH11_myoCAF, SPP1_Macro and MCs proportions in sample L2, estimated by CARD. **c** The spatial distribution of fibrosis scores in sample L1. **d** The spatial distribution of fibrosis scores in sample L2. **e** The spatial distribution of homotypic scores of VCAN_eCAF in sample L1. **f** The spatial distribution of homotypic scores of VCAN_eCAF in sample L2. **g** The spatial trajectory of fibrosis scores in sample L1, estimated by SPATA2. **h** The spatial trajectory of VCAN_eCAF proportions in sample L1, estimated by SPATA2. **i** The two-dimensional plots showing the changes in fibrosis scores and proportions of VCAN_eCAF, endothelial cells, MCs and MYH11_myoCAF along the spatial trajectory in sample L1, estimated by SPATA2. **j** The spatial trajectory of fibrosis scores in sample L2, estimated by SPATA2. **k** The spatial trajectory of VCAN_eCAF proportions in sample L2, estimated by SPATA2. **l** The two-dimensional plots showing the changes in fibrosis scores and proportions of VCAN_eCAF, endothelial cells, MCs and MYH11_myoCAF along the spatial trajectory in sample L2, estimated by SPATA2. **m** The spatial distribution of the *COL1A1*–*ITGB1* LR pair in sample L1. **n** The spatial distribution of the *COL1A1*–*ITGB1* LR pair in sample L2.

The combined analysis of single-cell RNA sequencing and ST data enabled the characterization of spatial patterns across diverse cell subpopulations within the TME. To investigate the spatial relationship between tumor fibrosis and specific cell types, we examined six cases of CRLM: L1, L2, 19G29, 19G61, 19G63 and 19G81 (Fig. 7 and Supplementary Figs. 11 and 12). Next, we used CARD’s algorithm to analyze spatial colocalization patterns of identified cell types across tissue sections (Supplementary Figs. 13a, 14a, 15a, 16a, 17a and 18a). We initially focused on cell types involved in COLLAGEN signaling interactions (MCs, MYH11_myoCAF and endothelial cells) through iTALK-based single-cell RNA sequencing analysis, as well as the fibrotic macrophage subset SPP1_Macro^65^, examining their spatial relationships with VCAN_eCAF. Our analysis revealed the consistent colocalization of VCAN_eCAF with MCs, MYH11_myoCAF and SPP1_Macro across all sections (Supplementary Figs. 13b, 14b, 15b, 16b, 17b and 18b). By contrast, the endothelial cells exhibited no stable association and were therefore excluded from further analysis. The spatial distribution maps via CARD of VCAN_eCAF with MCs, MYH11_MyoCAF and SPP1_Macro were further illustrated (Fig. 7a, b and Supplementary Figs. 11a, b and 12a, b). More intriguingly, in samples L1 and L2, we observed an overlap between the spatial distribution of tumor fibrosis scores and the proportion of VCAN_eCAF (Fig. 7a–d). The spatial organization of VCAN_eCAF, as assessed by homotypic scoring, mirrored the tumor fibrosis scores (Fig. 7e, f). Furthermore, SPATA2’s spatial trajectory analysis revealed that among all examined cell types (including SPP1_Macro, MYH11_myoCAF and MCs), VCAN_eCAF was the only population that exhibited concordant spatial variation with tumor fibrosis scores in terms of cellular abundance (Fig. 7g–i).

To validate the potential fibrosis-associated LR pairs identified via iTALK (Fig. 6i), we conducted the Spagene algorithm. We systematically evaluated multiple LR interactions (*COL1A1*–*ITGB1*, *COL4A5*–*ITGA2* and *COL5A2*–*DDR1*) across all tissue sections (Fig. 7 and Supplementary Figs. 11–18). Although the expression of *COL4A5*–*ITGA2* and *COL5A2*–*DDR1* was detectable, their spatial colocalization patterns, which were quantified by the LR score using the Spagene algorithm (Supplementary Figs. 13c, d, 14c, d, 15c, d16c, d, 17c, d and 18c, d), were negligible. By contrast, the *COL1A1*–*ITGB1* pair exhibited pronounced colocalization and visually overlapping spatial distributions with fibrosis scores (Fig. 7m, n and Supplementary Figs. 11m, n and 12m, n). Consistent with prior studies, *COL1A1* is the most abundant collagen in the ECM and is widely involved in pathological processes such as fibrosis, TME remodeling and tissue sclerosis, playing a critical role in diseases such as liver fibrosis and cancer metastasis^66^. *ITGB1*, as a core member of the integrin family, mediates cell–ECM adhesion and regulates cell migration, proliferation and survival^67^. Its aberrant expression is strongly associated with tumor invasion and fibrosis progression. Numerous studies have confirmed that the *COL1A1*–*ITGB1* signaling axis is a key pathway in ECM–cell interactions during fibrosis and cancer^68,69^, whereas other related pathways have relatively limited evidence. Collectively, these results provided spatial evidence supporting VCAN_eCAF’s involvement in tumor fibrosis via collagen-mediated signaling pathways within the TME of CRLM.

### Potential mechanism linking VCAN_eCAF to tumor fibrosis in Fibrosis+ LM

Historically, hepatic stellate cells have been recognized as the principal mediators of ECM synthesis in the liver, pivotal in the development of tumor fibrosis. Previous studies^70,71^ have consistently shown that tumor cells can induce hepatic stellate cell activation, leading to phenotypic transformations resembling ECM remodeling observed in VCAN_eCAF. To further explore this phenomenon, we conducted a comparative analysis of significantly upregulated LR pairs between VCAN_eCAF and MCs within the Fibrosis+ LM (Supplementary Fig. 19a). This analysis highlighted a notable upregulation of *FGFR1* in VCAN_eCAF, particularly within the Fibrosis+ LM (Supplementary Fig. 19b, c). Interestingly, the ligands for *FGFR1*, *FGF23* and *FGF3*, were specifically upregulated in MCs (Supplementary Fig. 19c–e). Based on these findings, we hypothesize that upon tumor cell metastasis to the liver, the secretion of fibroblast growth factors such as *FGF23* and *FGF3* may stimulate the phenotypic transformation of CAF precursor cells into VCAN_eCAF. Subsequently, VCAN_eCAF contributes to a TME characterized by tumor fibrosis, promoting the accumulation of suppressive T cells and profibrotic myeloid cells through the activation of the collagen signaling pathway.

## Discussion

This study investigates the clinical implications of tumor fibrosis in LM from patients with CRLM. We then dissected the TME heterogeneity of Fibrosis+ LM and Fibrosis− LM at the single-cell RNA sequencing level. Our key findings were validated through a range of multidimensional analyses, including ST and mIHC/IF analysis, all aimed at enhancing clinical decision-making.

Previous research has established a correlation between fibrosis and a T cell immunosuppressive milieu^7^. Our findings indicate a significant increase in classically immunosuppressive T cell subsets, notably CTLA4_CD4_T_reg_ and CXCL13_CD8_T_ex_. Moreover, our study identified an accumulation of proliferative T cell populations such as STMN1_CD8_T_pro_ and STMN1_CD4_T_pro_, which exhibit a stress-induced phenotype associated with immune checkpoint inhibition, potentially contributing to resistance against immunotherapy^16^. These observations suggest that the TME of Fibrosis+ LM presents a complex, multilayered T cell immunosuppressive environment. This complexity may hinder the direct enhancement of T cell-mediated antitumor responses, potentially leading to failures in systemic treatments and a higher rate of postoperative recurrence. Concurrently, our analysis of myeloid cells within the Fibrosis+ TME revealed a nuanced landscape. Previous studies have highlighted the fibrogenic properties of SPP1-positive macrophages^65^, findings consistent with our observation of a significant increase in SPP1_Macro within the Fibrosis+ LM. Together, these findings underscore the potent immunosuppressive characteristics of myeloid cells within the Fibrosis+ TME, which may impact both therapeutic strategies and patient outcomes adversely. Understanding these complex interactions between tumor cells, T cells and myeloid cells is crucial for developing effective immunotherapies and personalized treatment approaches for patients with Fibrosis+ LM.

Fibroblasts constitute a principal cellular component of the tumor stroma, demonstrating significant diversity and playing pivotal roles within the TME^72,73^. CAFs have been recognized as key drivers in the progression from tumor fibrosis to cirrhosis and HCC^74^. Studies have underscored the profibrotic phenotype of CAFs in cancer, contributing prominently to the establishment of a high-fibrotic TME^75,76^. Targeting these fibroblasts for antifibrotic therapies offers a promising strategy to reverse high-fibrotic TME, ultimately enhancing treatment efficacy and improving patient prognosis^77^. In this study, we identified a specific subgroup of CAFs characterized by high expression of *VCAN*. These CAFs also exhibited elevated levels of ECM-related genes, including *LUM*, *COL1A1* and *COL1A2*, indicative of their activated state promoting tumor fibrosis. Notably, this subgroup of fibroblasts showed heightened responsiveness to the Fibrosis+ as a biological variable. A detailed analyses of cellular communication suggested that their activation played a pivotal role in reshaping the TME within the Fibrosis+ LM. Furthermore, our investigation into collagen signaling pathways implicated their regulatory influence on ligands interacting with T cells and myeloid cells within the TME, potentially exacerbating fibrosis. The spatial analysis revealed a colocalization of these *VCAN*-expressing CAFs with regions of intense tumor fibrosis, indicating their potential contribution to the high-fibrotic phenotype of the TME in Fibrosis+ LM. The *FGFR* signaling pathway, crucial for cellular processes such as proliferation and differentiation, has been implicated in various cancers^78^. Previous studies have highlighted the role of *FGF*–*FGFR* signaling in promoting fibrosis, with extensive expression observed at fibrotic sites^71,79,80^. Furthermore, we found that Fibrosis+ LM-derived MCs exhibit the specific upregulation of the Wnt signaling pathway. Previous literature has reported a correlation between ECM remodeling and the Wnt pathway. For instance, Liu et al.^81^ found that pancreatic cancer cells can sense ECM stiffness and activate the Wnt–β-catenin–*TCF4* signaling pathway, leading to the upregulation of *CLIC1* expression and ultimately promoting glycolysis-dependent tumor growth. Penny et al.^82^ constructed a minimally invasive orthotopic xenograft model to demonstrate that targeting the oncogenic Wnt–β-catenin signaling pathway disrupts ECM expression and inhibits the growth of adrenal cortical carcinoma. The study further validated the effective control of tumor burden by inhibiting the Wnt–β-catenin axis using the novel small molecule inhibitor Tegavivint. Zhang et al.^83^ reported that fibroblast growth factors may accelerate liver fibrosis by positively regulating Wnt signaling, which increases the production of ECM. The resulting increase in liver fibrosis could be a carcinogenic mechanism by which fibroblast growth factors promote NASH-driven HCC. Our study observed an increase in the interaction between *FGF23* or *FGF3* and *FGFR1* receptor-ligands between MCs and *VCAN*-expressing CAFs, suggesting a mechanism by which MCs secrete fibroblast growth factors that enhance the activation of *VCAN*-expressing CAFs, thereby influencing the TME in Fibrosis+ LM. This suggests that targeting VCAN_eCAF may improve prognosis by remodeling the fibrotic microenvironment and inhibiting tumor cell responses to ECM remodeling, with the underlying mechanisms warranting further investigation.

Several limitations of the present study warrant consideration. First, its retrospective design precludes the establishment of causal relationships between variables, as confounding factors may have influenced the observed results. Future prospective cohort studies are essential to validate our findings more robustly. Second, challenges in obtaining an adequate number of single-cell samples may have introduced bias in the identification of cell types and transitional pathways.

In summary, this study utilized clinical data to establish a correlation between tumor fibrosis in LM and poor survival outcomes in patients with CRLM. Moreover, it preliminarily explored potential mechanisms underlying the formation of tumor fibrosis, highlighting a promising direction for targeting VCAN_eCAF in this patient population.

## Supplementary information

Supplementary Information

Supplementary Table

## Data Availability

The datasets generated during and/or analyzed during the current study are available from the corresponding author (H.Z., pumc95zhao@126.com) on reasonable request.
