# Supplementary material for: Single-cell transcriptomic profiling reveals liver fibrosis in colorectal cancer liver metastasis
Source: Exp Mol Med. 2025 Nov 14;57(11):2517–32. doi: 10.1038/s12276-025-01573-3 (PMC12686428; doi:10.1038/s12276-025-01573-3)
Supplement: Supplementary file 1 — Supplementary Information [file 12276_2025_1573_MOESM1_ESM.pdf]

Supplementary Materials

Quality Assurance Procedures for Single-Cell RNA-Seq Data

Single-cell gel bead-in-emulsion (GEMs) production was conducted using the 10x Genomics Chromium Controller (version 3) with suspensions of individual cells. Following manufacturer protocols, mRNA molecules encapsulated within droplets underwent reverse-transcription reactions to synthesize and amplify cDNAs. Subsequently, the resulting 10x libraries were sequenced on the NovaSeq platform (Illumina, San Diego, CA). Alignment of the sequencing data to the GRCh38 human reference genome was performed using the Cell Ranger toolkit (version 2.1.0).

Initially, cells of insufficient quality were filtered based on three criteria: 1) exclusion of cells with fewer than 3 detected genes; 2) exclusion of cells with fewer than 50 detected genes in total; and 3) removal of cells where mitochondrial genes constituted 20% or more, or hemoglobin genes constituted 5% or more, of the total gene expression. Following quality control, data processing utilized the Seurat R package v4.0.2. Batch effects among different samples were mitigated and dimensionality reduction was performed using the harmony [1] function. Normalized counts were obtained using the SCTransform [2] function, which included mitochondrial gene percentage regression. The optimal number of principal components was determined using the ElbowPlot function in Seurat.

Subsequently, cells were clustered using the sharing nearest neighbor (SNN) modularity optimization algorithm, and the Uniform Manifold Approximation and Projection (UMAP) algorithm was employed to project all cells into a

two-dimensional space for visualization. Cell identity assignment to each cluster relied on highly expressed genes, uniquely expressed genes, and established cellular markers.

Analysis of Differential Gene Expression

To pinpoint differentially expressed genes across samples, the Function FindMarkers was employed with the parameter ‘min.pct = .25, logfc.threshold = .25’, harnessing the Wilcoxon rank sum test algorithm.

Assessment of Copy Number Variations

Chromosomal copy number variations (CNVs) were estimated using the R package inferCNV. CNV scores were computed by summing the CNV levels across cells within each subcluster.

Multiplex immunohistochemistry

FFPE tissue slides of 3um thickness underwent deparaffinization and rehydration using a gradient of alcohol. The antigen epitope retrieval solution, containing citrate buffer at pH 9.0, was preheated. Following this, the slides were incubated with 3% H2O2 for 20 minutes to quench endogenous peroxidase activity. Subsequently, a pre-incubation step with 10% normal goat serum for 20 minutes was followed by a 30-minute incubation with primary antibodies from four panels: CD3, CD8, and CXCL13; CD3, CD4, and CTLA4; CD68 and SPP1; and DCN and VCAN. HRP-conjugated secondary antibodies and fluorescent dyes corresponding to each antibody (provided by Abcarta Inc.) were applied. Imaging and scanning were conducted using the KF-PRO-020 digital pathology slide scanner (KFBIO Inc.). The

resulting kfbf files were directly imported into HALO® software (Version 3.0; Indica Labs, Albuquerque, New Mexico, USA; accessible at https://indicalab.com/halo/) for quantitative image analysis.

The analysis encompassed two main steps: (1) Cell Categorization: Cells were initially identified and segmented based on nuclear DAPI signaling. Using nine primary fluorescence channels (CD3, CD4, CD8, CXCL13, CTLA4, CD68, SPP1, DCN, and VCAN), four distinct cell types were defined: CTLA4+ CD4 Treg (positive for CD3, CD4, and CTLA4), CXCL13+ CD8 Tex (positive for CD3, CD8, and CXCL13), SPP1+ macrophages (positive for SPP1 and CD68), and VCAN+ cancer-associated fibroblasts (positive for DCN and VCAN). (2) Detection: The Highlex FL module was utilized for simultaneous detection of various cell types. Signal intensity quality in each slide was evaluated by two pathologists (BW and JL). Following module execution, summary data for each slide were exported for further analysis.

Reference

1. Korsunsky I, et al.. Fast, sensitive and accurate integration of single-cell data with Harmony. Nat Methods (2019) 16(12):1289-96.

2. Hafemeister C, Satija R. Normalization and variance stabilization of single-cell RNA-seq data using regularized negative binomial regression. Genome Biol (2019) 20(1):296.

Supplementary figure legends:

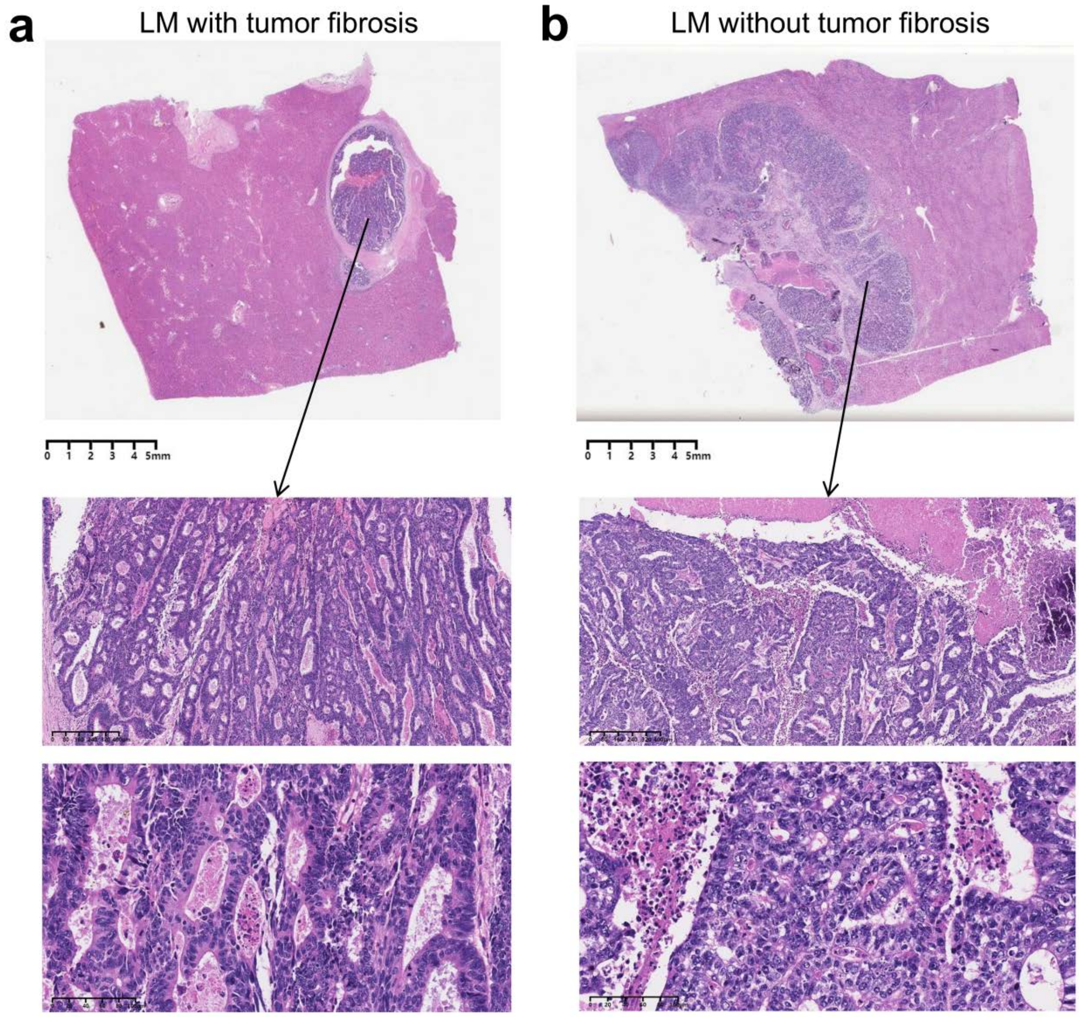

Supplementary Fig. 1. Fibrosis pathological assessment of liver metastases in CRLM. **a-b** Representative H&E staining of CRLM with **(a)** an without **(b)** tumor fibrosis.

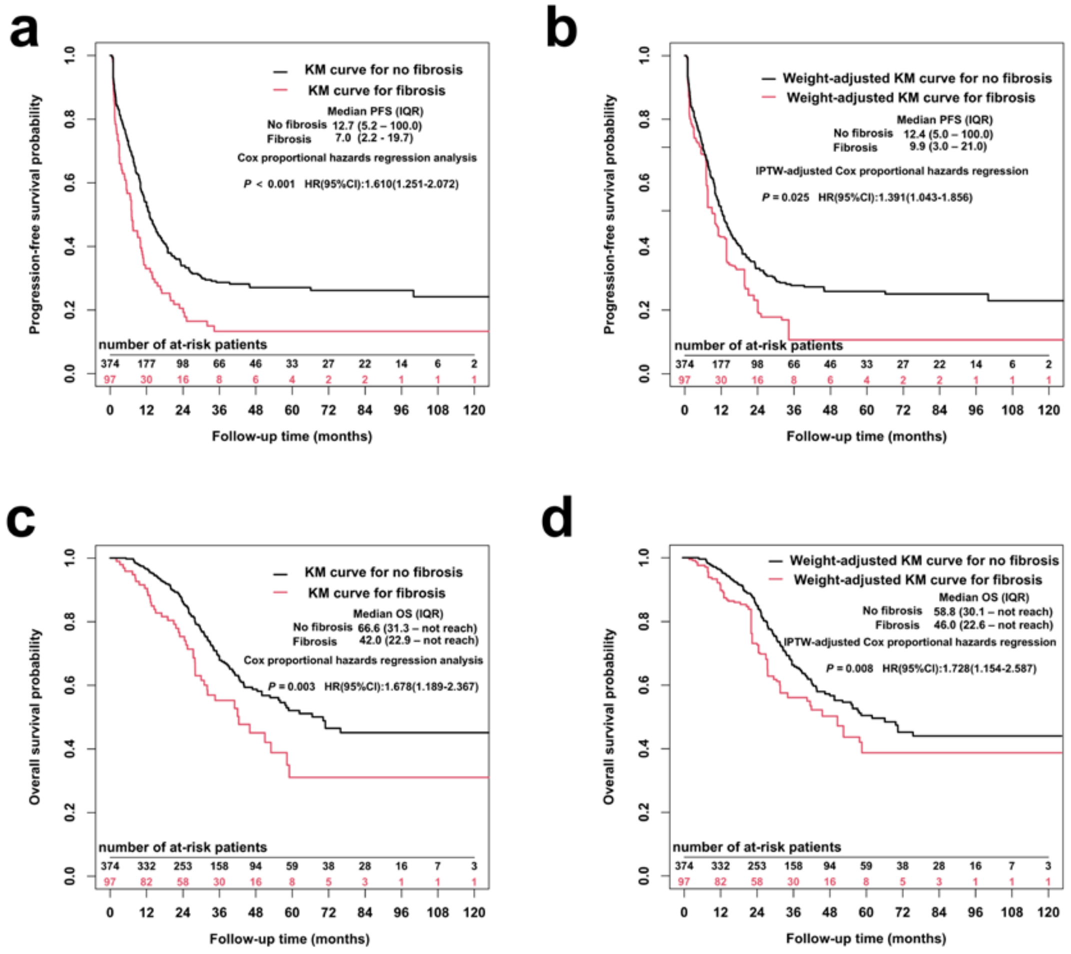

Supplementary Fig. 2. Survival analysis of CRLM patients with Fibrosis- LM versus Fibrosis+ LM. **a** KM curve of PFS before IPTW adjustment. **b** KM curve of PFS after IPTW adjustment. **c** KM curve of OS before IPTW adjustment. **d** KM curve of OS after IPTW adjustment.

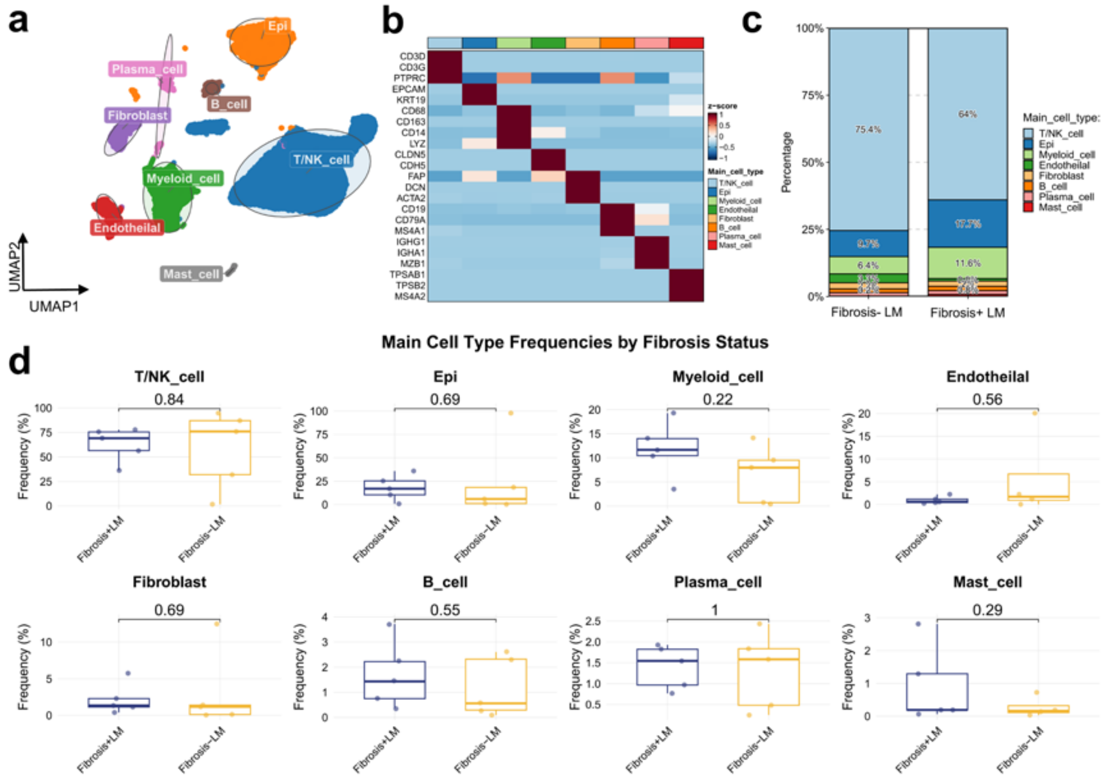

Supplementary Fig. 3. The global analysis of single cell sequencing. **a** UMAP plot showing all the main cell clusters. **b** The heatmap showing marker genes of annotated cell clusters. **c** The bar plot showing the proportion of the main cell clusters between Fibrosis+ LM and Fibrosis- LM. **d** Box plots comparing the proportions of eight main cell clusters between fibrosis-positive (Fibrosis+) and fibrosis-negative (Fibrosis–) groups (Wilcoxon signed-rank test).

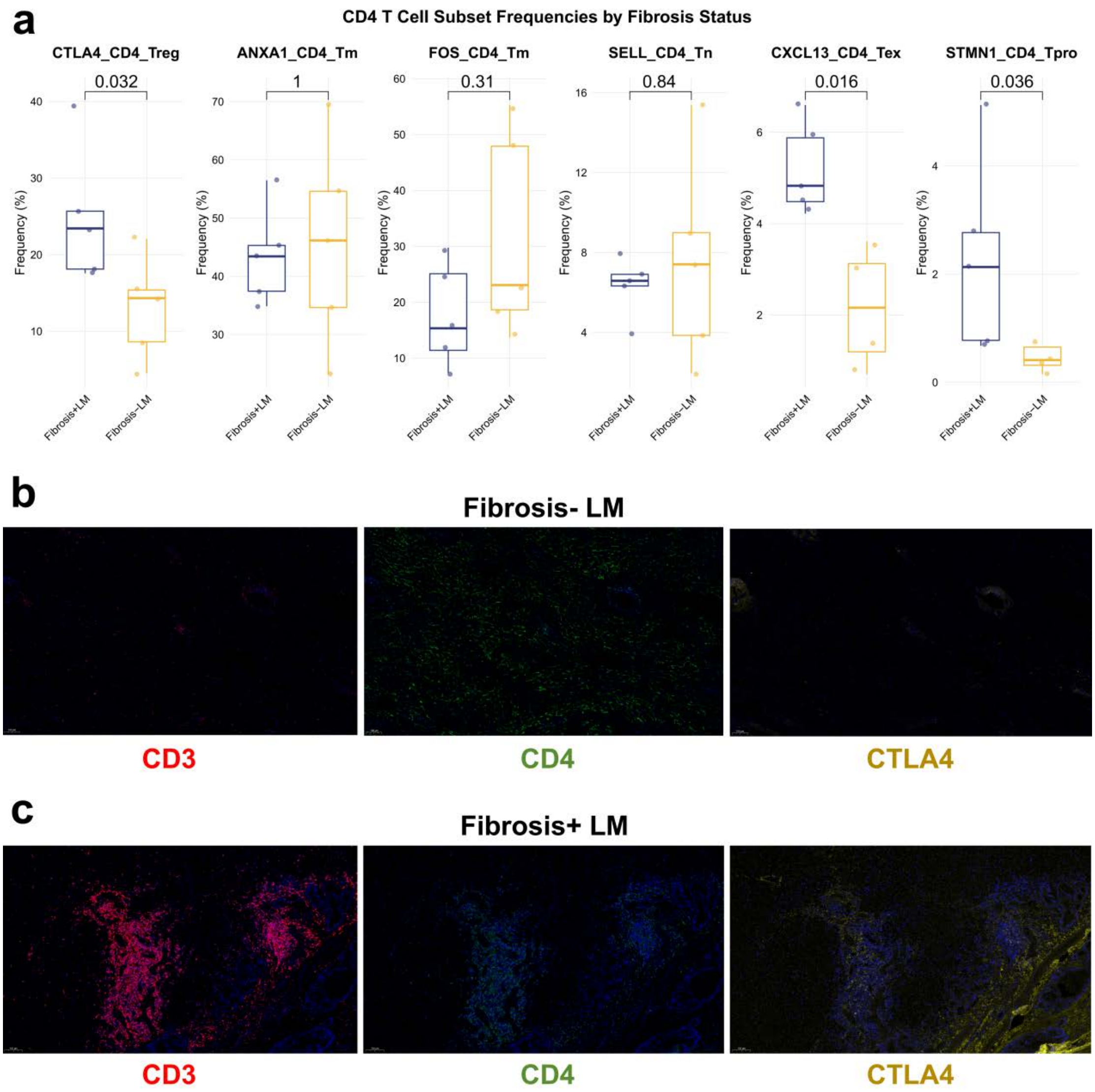

Supplementary Fig. 4. Additional characterization of $CD4^+$ T cell subsets in this study. **a** Box plots comparing the proportions of six $CD4^+$ T cell subsets between fibrosis-positive (Fibrosis+) and fibrosis-negative (Fibrosis–) groups (Wilcoxon signed-rank test). **b** Representative immunofluorescence images showing single-marker expression of CD3, CD4, and CTLA4 in Fibrosis– tissues. **c** Representative immunofluorescence images showing single-marker expression of CD3, CD4, and CTLA4 in Fibrosis+ tissues.

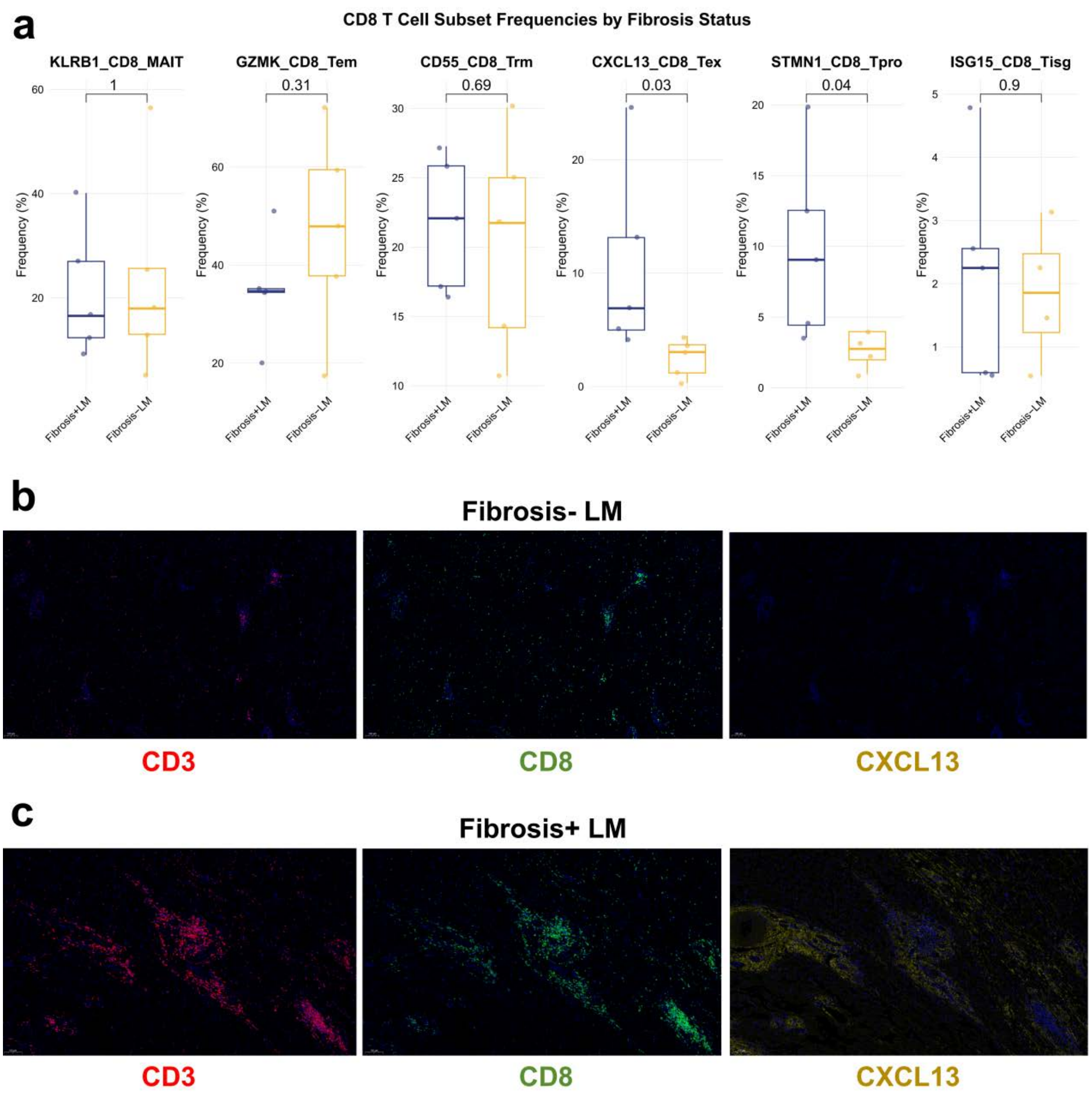

Supplementary Fig. 5. Additional characterization of CD8$^+$ T cell subsets in this study. **a** Box plots comparing the proportions of six CD8$^+$ T cell subsets between fibrosis-positive (Fibrosis+) and fibrosis-negative (Fibrosis–) groups (Wilcoxon signed-rank test). **b** Representative immunofluorescence images showing single-marker expression of CD3, CD8, and CXCL13 in Fibrosis– tissues. **c** Representative immunofluorescence images showing single-marker expression of CD3, CD8, and CXCL13 in Fibrosis+ tissues.

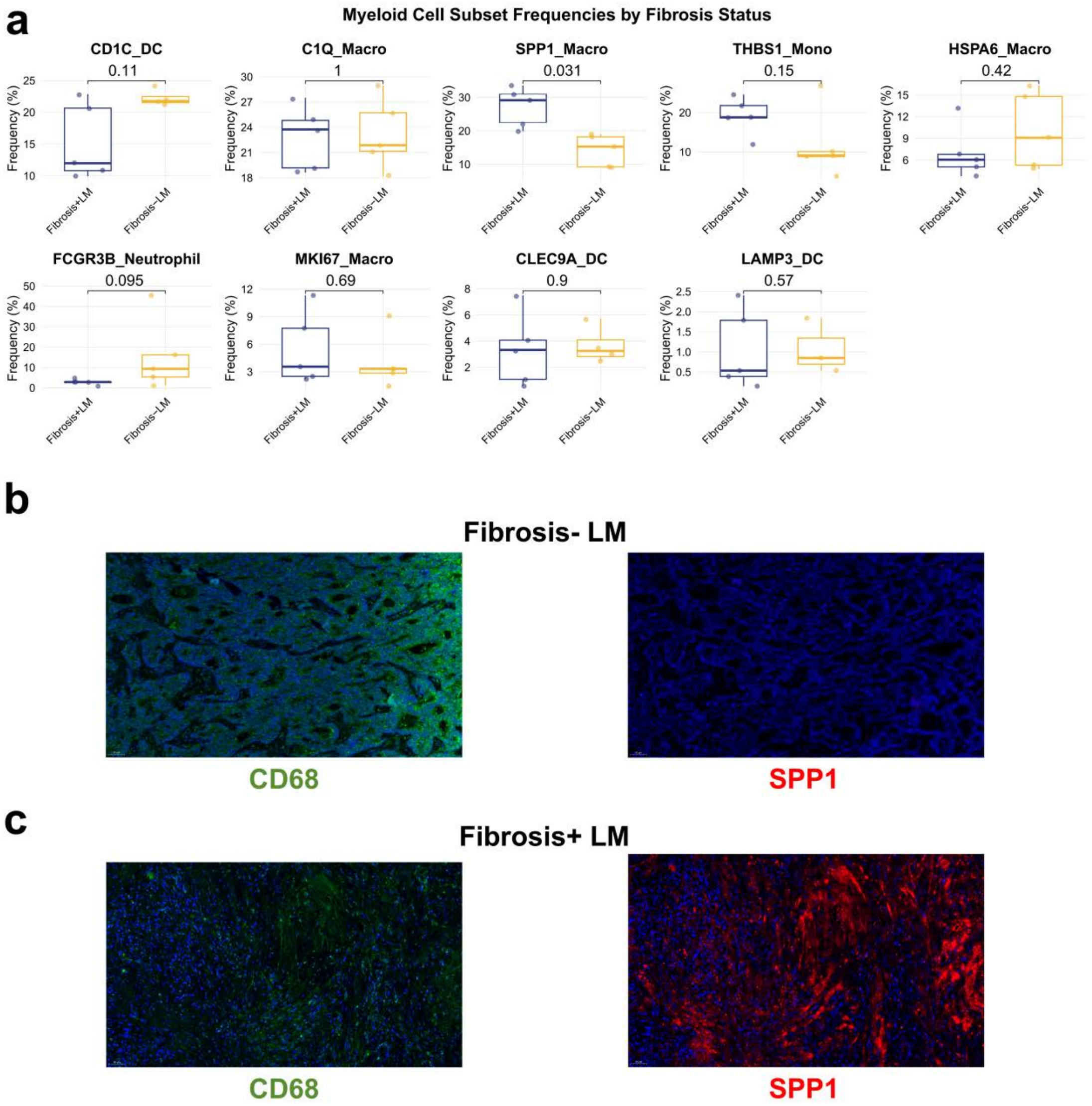

Supplementary Fig. 6. Additional characterization of myeloid cell subsets in this study. **a** Box plots comparing the proportions of nine myeloid cell subsets between fibrosis-positive (Fibrosis+) and fibrosis-negative (Fibrosis–) groups (Wilcoxon signed-rank test). **b** Representative immunofluorescence images showing single-marker expression of CD68, and SPP1 in Fibrosis– tissues. **c** Representative immunofluorescence images showing single-marker expression of CD68, and SPP1 in Fibrosis+ tissues.

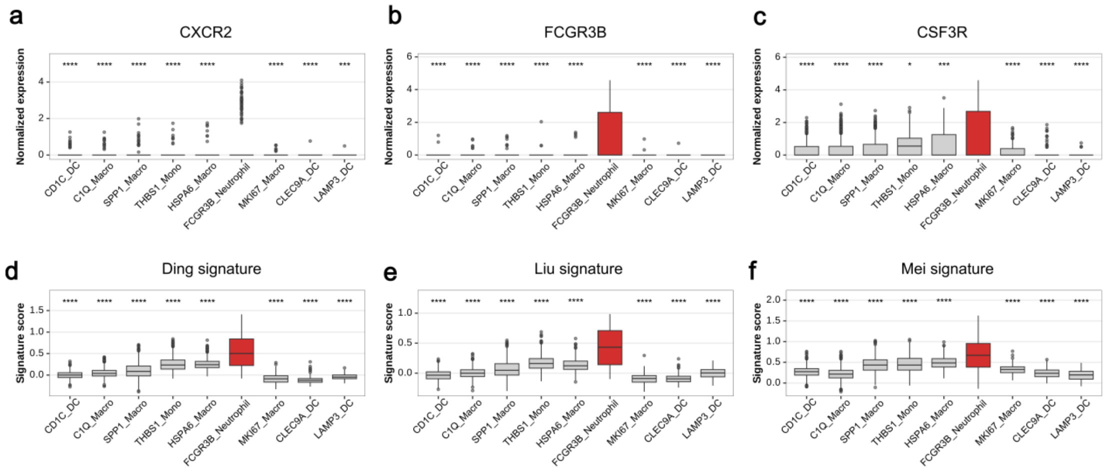

Supplementary Fig. 7. Additional characterization of neutrophils in this study. **a-c** Box plots comparing the normalized expression levels of CXCR2 (**a**), FCGR3B (**b**), and CSF3R (**c**) between neutrophils and other myeloid cell subsets (Wilcoxon signed-rank test). **d-f** Validation of neutrophil signature scores derived from three independent gene sets across different myeloid cell types (Wilcoxon signed-rank test).

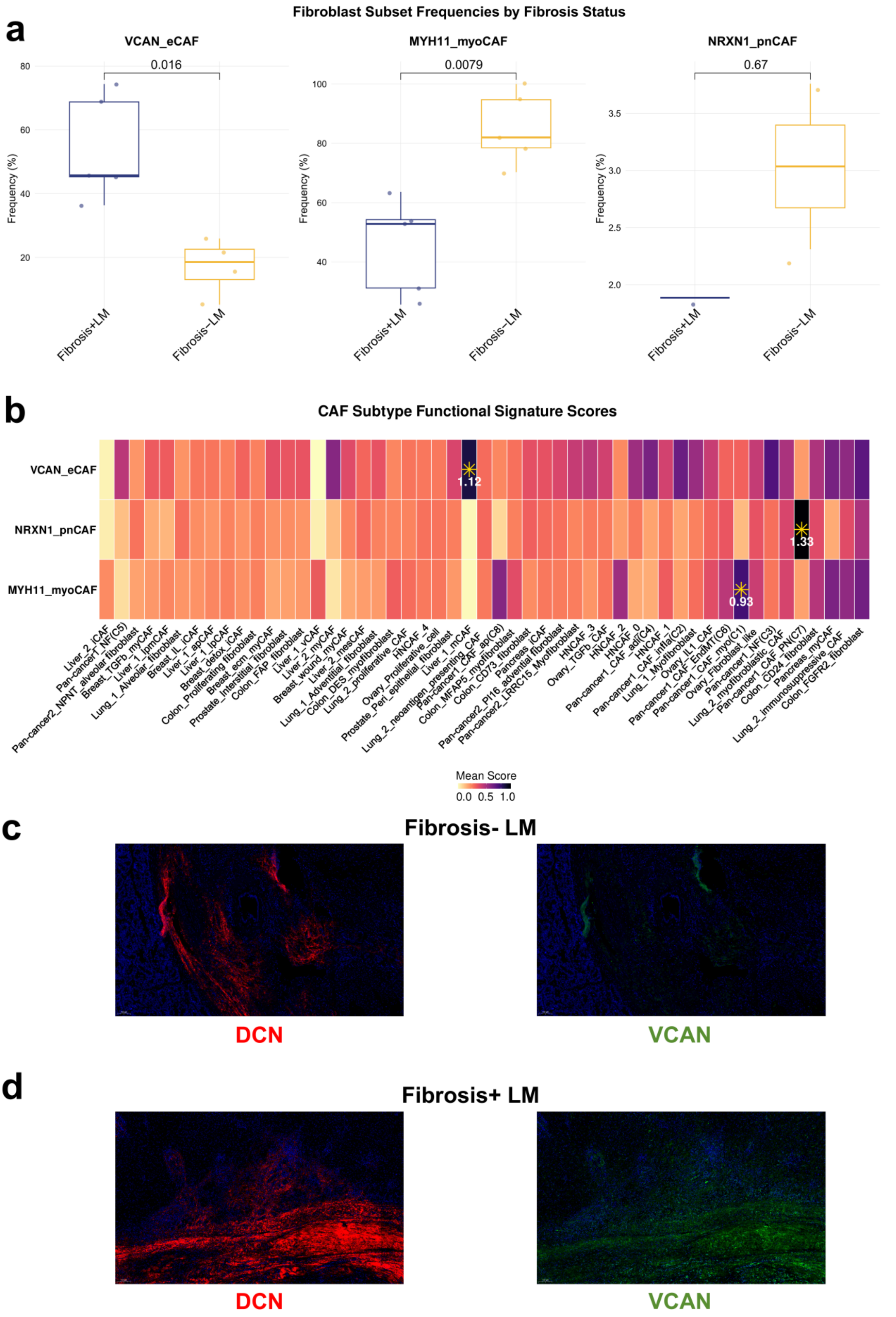

Supplementary Fig. 8. Additional characterization of CAF in this study. **a** Box plots comparing the proportions of three CAF subsets between fibrosis-positive (Fibrosis+) and fibrosis-negative (Fibrosis–) groups (Wilcoxon signed-rank test). **b** Heatmap

displaying the enrichment scores of all CAF signature genes identified from 11 independent CAF classification studies across the three CAF subtypes characterized in this study. Asterisks denote the highest-scoring subtypes, with color intensity positively correlating with enrichment scores (darker colors indicate higher scores). **c** Representative immunofluorescence images showing single-marker expression of DCN, and VCAN in Fibrosis– tissues. **d** Representative immunofluorescence images showing single-marker expression of DCN, and VCAN in Fibrosis+ tissues.

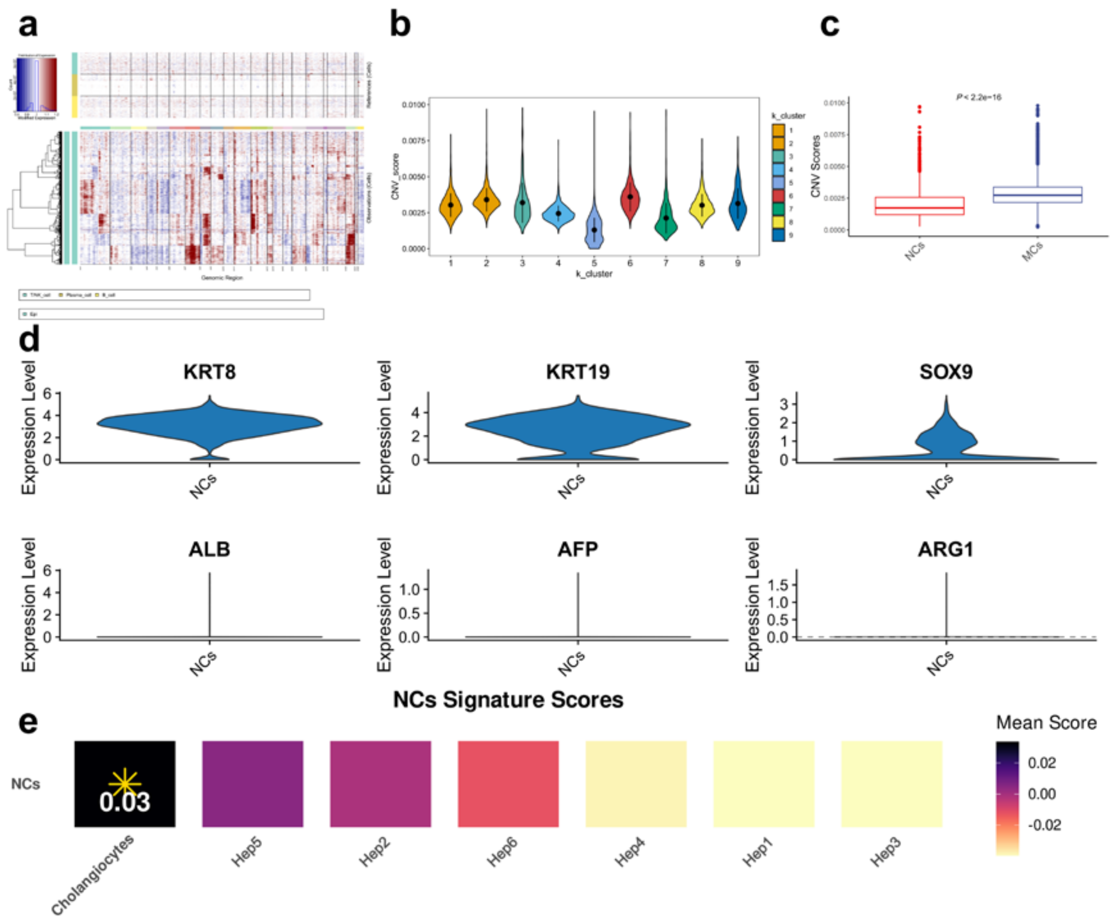

Supplementary Fig. 9. Additional characterization of epithelial cells in this study. **a-c** The process of selecting malignant cells (MCs): Heatmap showing an overall CNV plot (**a**); Clustering of CNV patterns into groups 1-9, revealing clusters 5 and 7 with significantly lower scores compared to others **(b)**; The boxplot compares CNV scores between annotated MCs and normal cells (NCs) (**c**), Wilcox rank-sum test. **d** Violin plots showing expression levels of cholangiocyte markers (KRT19, SOX9, KRT7) and hepatocyte markers (ALB, HNF4A, ABCC2) in NCs. **e** Heatmap showing enrichment

scores of cholangiocyte signature and six hepatocyte subtype signatures in NCs with asterisks indicating the highest-scoring subtypes (darker colors represent higher enrichment scores).

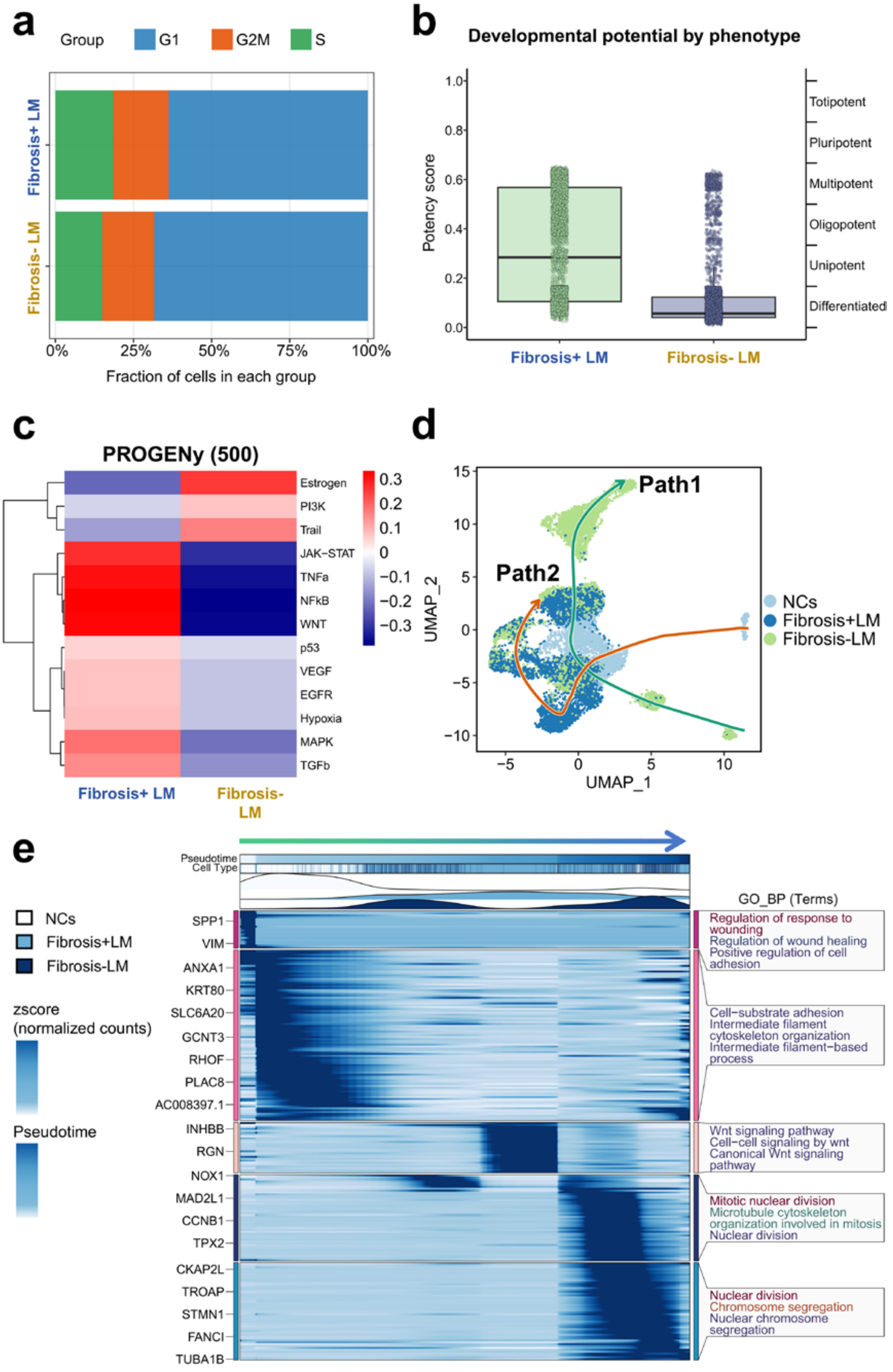

Supplementary Fig. 10. Additional analysis of malignant cells (MCs). **a** A stacked plot showing the proportion of cells in G1, S, and G2M phases in Fibrosis+LM

derived MCs and Fibrosis-LM derived MCs. The colors represent different cell cycle phases. **b** A box plot depicting the stemness levels calculated by Cytotrace in Fibrosis+LM derived MCs (left) and Fibrosis-LM derived MCs (right). **c** A heatmap illustrating the diverse PROGENy activity scores for Fibrosis+LM derived MCs (left) and Fibrosis-LM derived MCs (right), based on normalized gene signature scores. Deeper red hues indicate higher enrichment levels. **d** A UMAP plot showing the developmental trajectory identified by Slingshot, with arrows indicating the direction of pseudotime trajectory. Different cell types are represented by distinct colors. **e** A heatmap showing the dynamic DEGs and their enriched pathways along the pseudotime trajectory transitioning from NCs to Fibrosis+LM derived MCs and Fibrosis-LM derived MCs. These DEGs were divided into five main clusters.

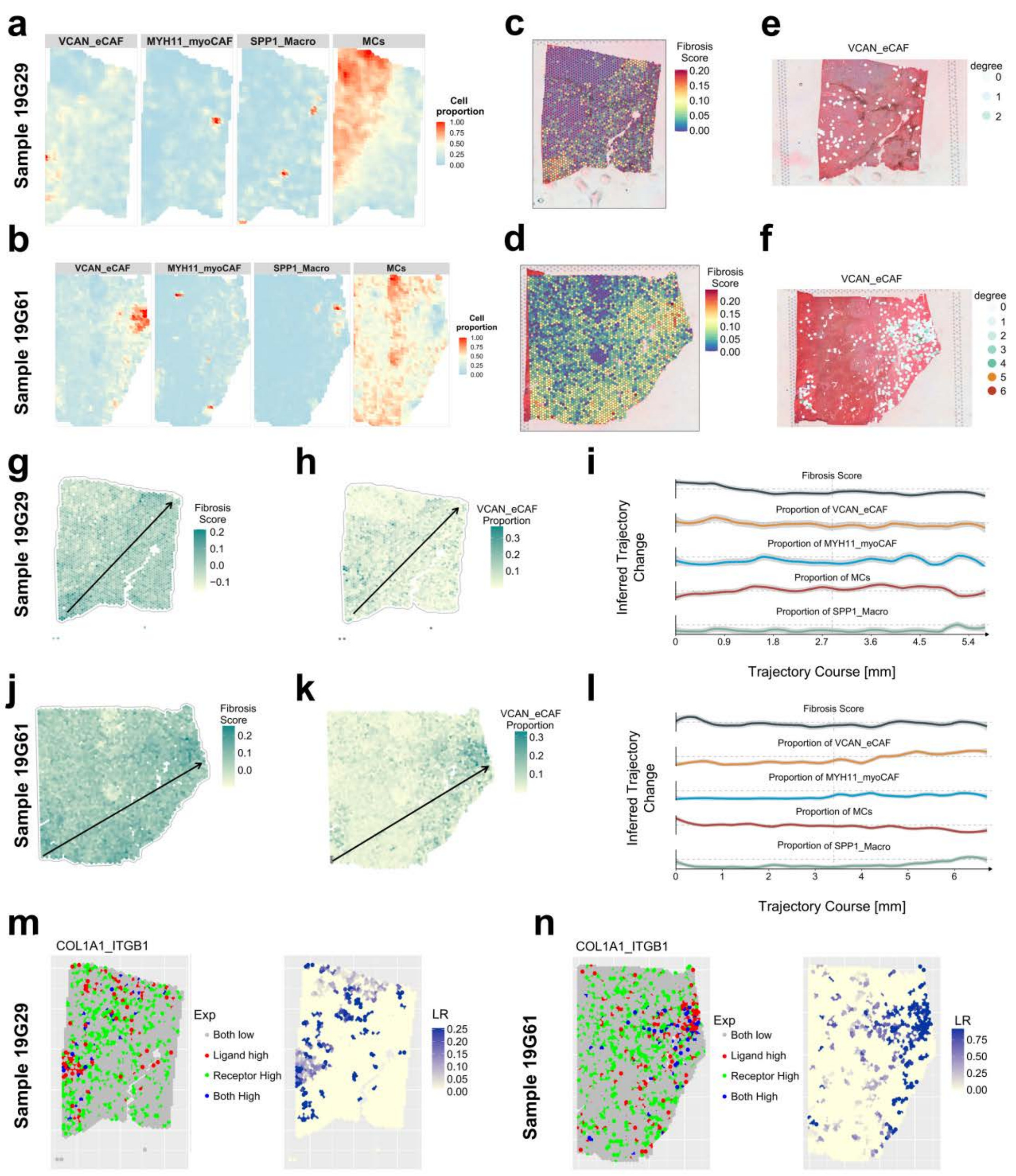

Supplementary Fig. 11. The relationship between VCAN_eCAF and tumor fibrosis was assessed using spatial RNA-seq in sample 19G29 and 19G61. **a** Spatial distribution of VCAN_eCAF, MYH11_myoCAF, SPP1_Macro and MCs proportions in sample 19G29, estimated by CARD. **b** Spatial distribution of fibrosis scores in sample 19G29. **c** Spatial distribution of VCAN_eCAF, MYH11_myoCAF, SPP1_Macro and MCs proportions in sample 19G61, estimated by CARD. **d** Spatial distribution of fibrosis scores in sample 19G61. **e** Spatial distribution of homotypic scores of VCAN_eCAF in sample 19G29. **f** Spatial distribution of homotypic scores

of VCAN_eCAF in sample 19G61. **g** Spatial trajectory of fibrosis scores in sample 19G29, estimated by SPATA2. **h** Spatial trajectory of VCAN_eCAF proportions in sample 19G29, estimated by SPATA2. **i** Two-dimensional plots showing the changes in fibrosis scores and proportions of VCAN_eCAF, endothelial cells, MCs, and MYH11_myoCAF along the spatial trajectory in sample 19G29, estimated by SPATA2. **j** Spatial trajectory of fibrosis scores in sample 19G61, estimated by SPATA2. **k** Spatial trajectory of VCAN_eCAF proportions in sample 19G61, estimated by SPATA2. **l** Two-dimensional plots showing the changes in fibrosis scores and proportions of VCAN_eCAF, endothelial cells, MCs, and MYH11_myoCAF along the spatial trajectory in sample 19G61, estimated by SPATA2. **m** Spatial distribution of the COL1A1-ITGB1 ligand-receptor pair in sample 19G29. **n** Spatial distribution of the COL1A1-ITGB1 ligand-receptor pair in sample 19G61.

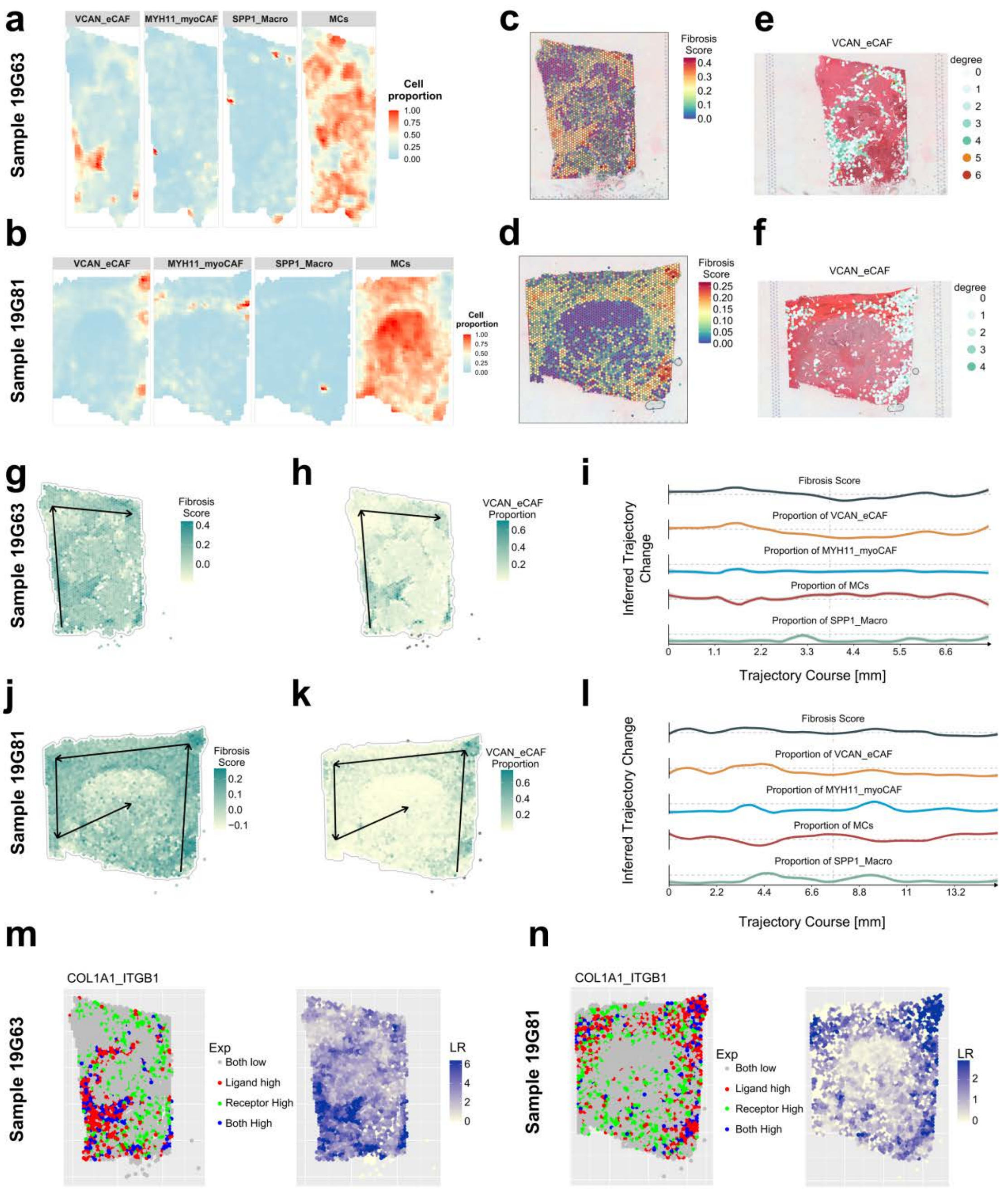

Supplementary Fig. 12. The relationship between VCAN_eCAF and tumor fibrosis was assessed using spatial RNA-seq in sample 19G63 and 19G81. **a** Spatial distribution of VCAN_eCAF, MYH11_myoCAF, SPP1_Macro and MCs proportions in sample 19G63, estimated by CARD. **b** Spatial distribution of fibrosis scores in sample 19G81. **c** Spatial distribution of VCAN_eCAF, MYH11_myoCAF, SPP1_Macro and MCs proportions in sample 19G63, estimated by CARD. **d** Spatial distribution of fibrosis scores in sample 19G81. **e** Spatial distribution of homotypic scores of VCAN_eCAF in sample 19G63. **f** Spatial distribution of homotypic scores

of VCAN_eCAF in sample 19G81. **g** Spatial trajectory of fibrosis scores in sample 19G63, estimated by SPATA2. **h** Spatial trajectory of VCAN_eCAF proportions in sample 19G63, estimated by SPATA2. **i** Two-dimensional plots showing the changes in fibrosis scores and proportions of VCAN_eCAF, endothelial cells, MCs, and MYH11_myoCAF along the spatial trajectory in sample 19G63, estimated by SPATA2. **j** Spatial trajectory of fibrosis scores in sample 19G81, estimated by SPATA2. **k** Spatial trajectory of VCAN_eCAF proportions in sample 19G81, estimated by SPATA2. **l** Two-dimensional plots showing the changes in fibrosis scores and proportions of VCAN_eCAF, endothelial cells, MCs, and MYH11_myoCAF along the spatial trajectory in sample 19G81, estimated by SPATA2. **m** Spatial distribution of the COL1A1-ITGB1 ligand-receptor pair in sample 19G63. **n** Spatial distribution of the COL1A1-ITGB1 ligand-receptor pair in sample 19G81.

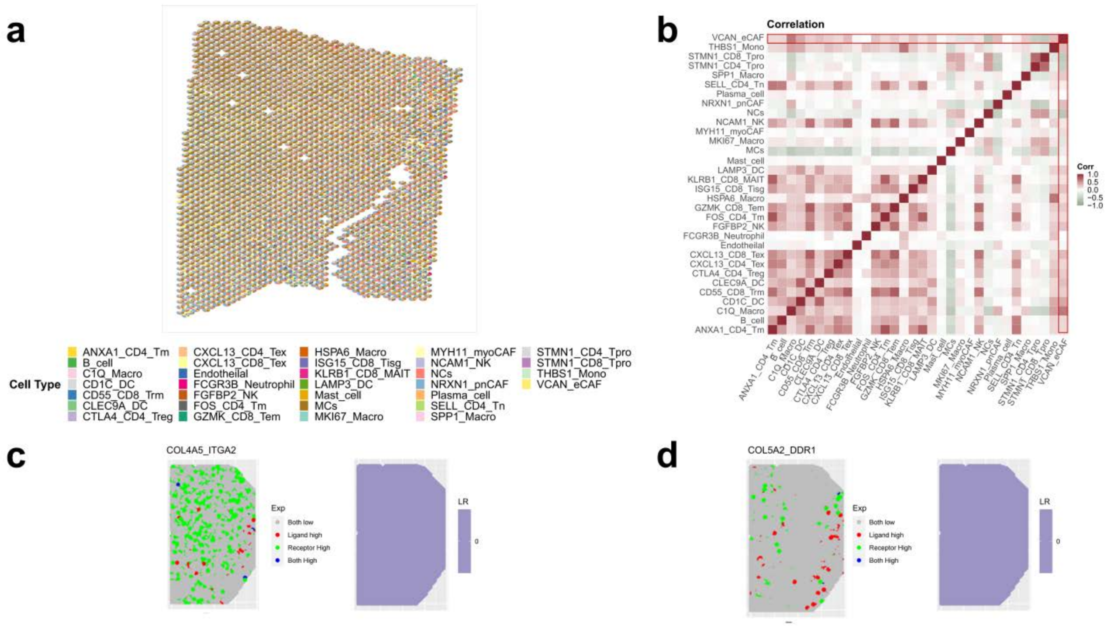

Supplementary Fig. 13. Spatial transcriptomic profiling of sample L1. **a** Cell-type deconvolution analysis performed using CARD method reveals spatially resolved cellular composition. **b** Correlation matrix of cell type proportions across spatial locations inferred by CARD, with color gradient representing correlation coefficients. **c-d** Spatial distribution patterns of ligand-receptor pairs: COL4A5-ITGA2 **(c)** and COL5A2-DDR1 **(d)** interactions in sample L1.

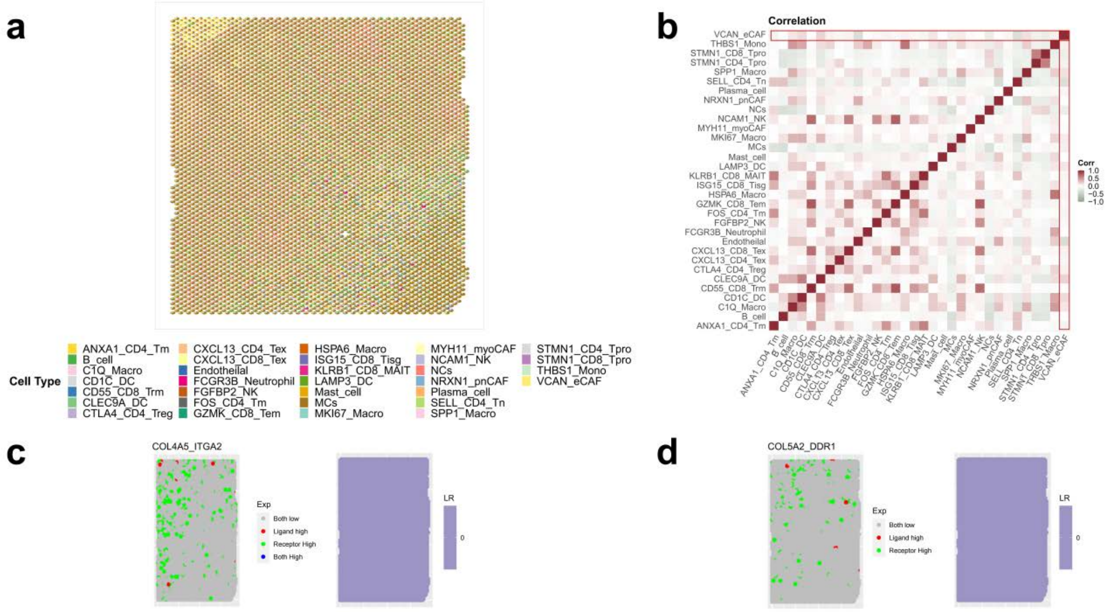

Supplementary Fig. 14. Spatial transcriptomic profiling of sample L2. **a** Cell-type deconvolution analysis performed using CARD method reveals spatially resolved cellular composition. **b** Correlation matrix of cell type proportions across spatial locations inferred by CARD, with color gradient representing correlation coefficients. **c-d** Spatial distribution patterns of ligand-receptor pairs: COL4A5-ITGA2 **(c)** and COL5A2-DDR1 **(d)** interactions in sample L2.

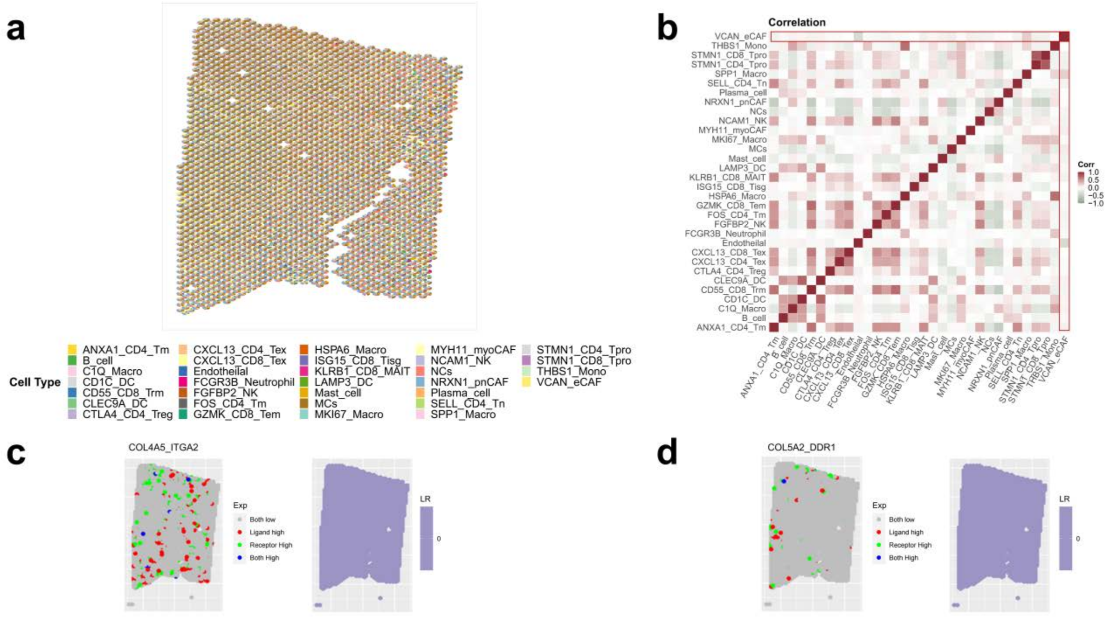

Supplementary Fig. 15. Spatial transcriptomic profiling of sample 19G29. **a** Cell-type deconvolution analysis performed using CARD method reveals spatially resolved cellular composition. **b** Correlation matrix of cell type proportions across spatial locations inferred by CARD, with color gradient representing correlation coefficients. **c-d** Spatial distribution patterns of ligand-receptor pairs: COL4A5-ITGA2 **(c)** and COL5A2-DDR1 **(d)** interactions in sample 19G29.

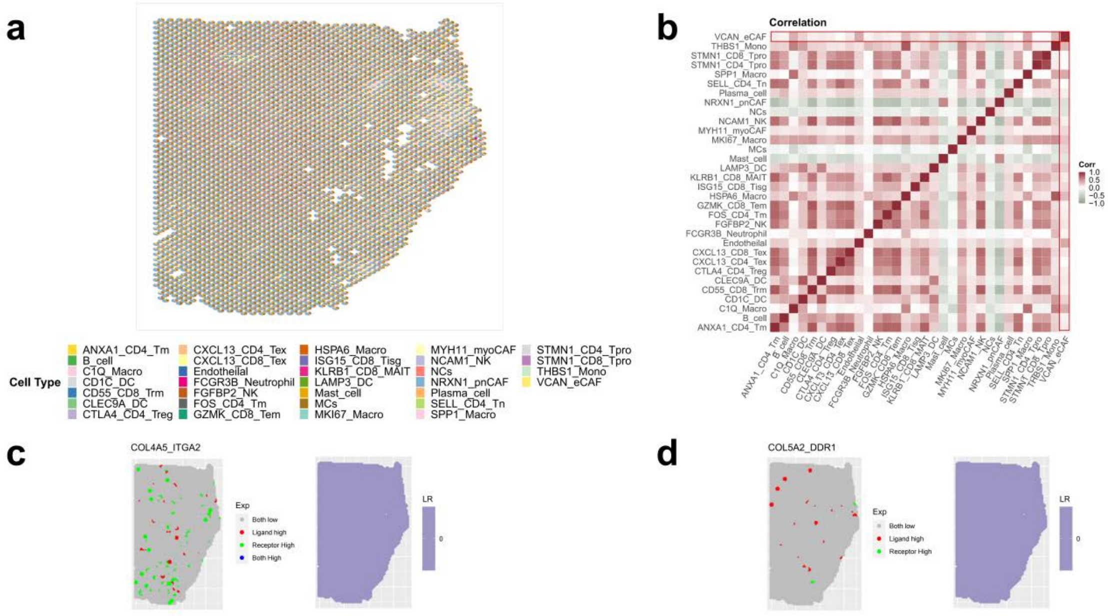

Supplementary Fig. 16. Spatial transcriptomic profiling of sample 19G61. **a**

Cell-type deconvolution analysis performed using CARD method reveals spatially resolved cellular composition. **b** Correlation matrix of cell type proportions across spatial locations inferred by CARD, with color gradient representing correlation coefficients. **c-d** Spatial distribution patterns of ligand-receptor pairs: COL4A5-ITGA2 **(c)** and COL5A2-DDR1 **(d)** interactions in sample 19G61.

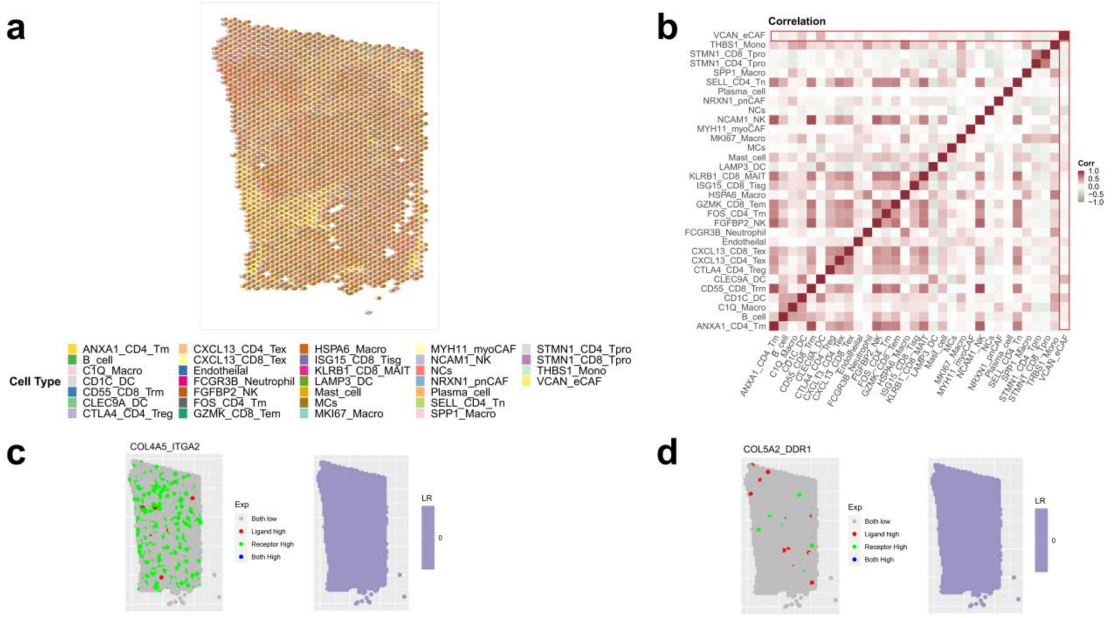

Supplementary Fig. 17. Spatial transcriptomic profiling of sample 19G63. **a** Cell-type deconvolution analysis performed using CARD method reveals spatially resolved cellular composition. **b** Correlation matrix of cell type proportions across spatial locations inferred by CARD, with color gradient representing correlation coefficients. **c-d** Spatial distribution patterns of ligand-receptor pairs: COL4A5-ITGA2 **(c)** and COL5A2-DDR1 **(d)** interactions in sample 19G63.

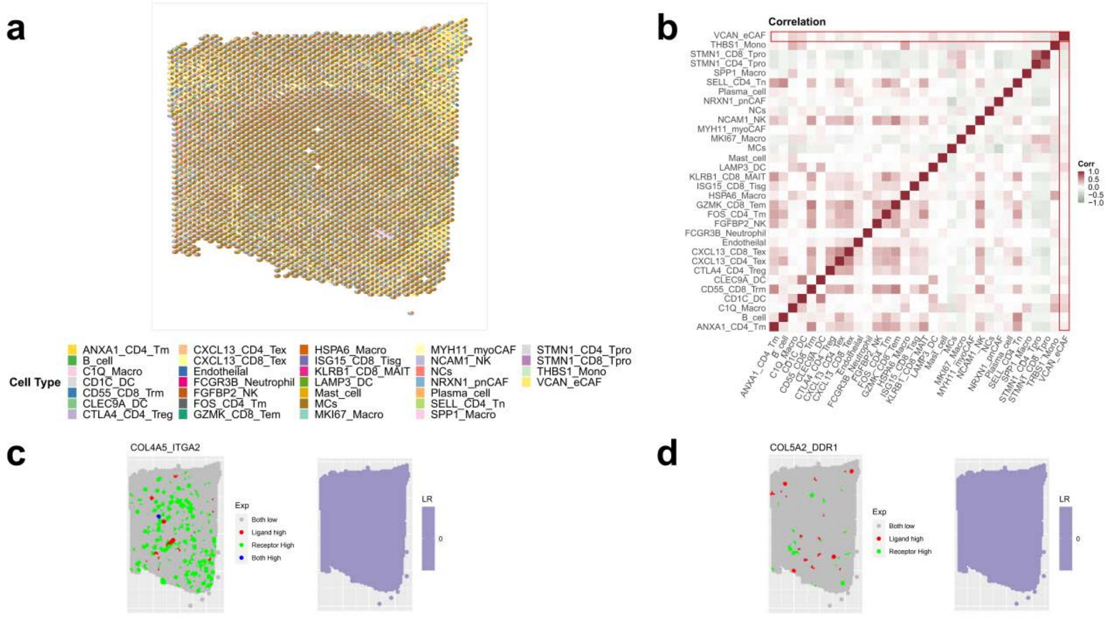

Supplementary Fig. 18. Spatial transcriptomic profiling of sample 19G81. **a** Cell-type deconvolution analysis performed using CARD method reveals spatially resolved cellular composition. **b** Correlation matrix of cell type proportions across spatial locations inferred by CARD, with color gradient representing correlation coefficients. **c-d** Spatial distribution patterns of ligand-receptor pairs: COL4A5-ITGA2 **(c)** and COL5A2-DDR1 **(d)** interactions in sample 19G81.

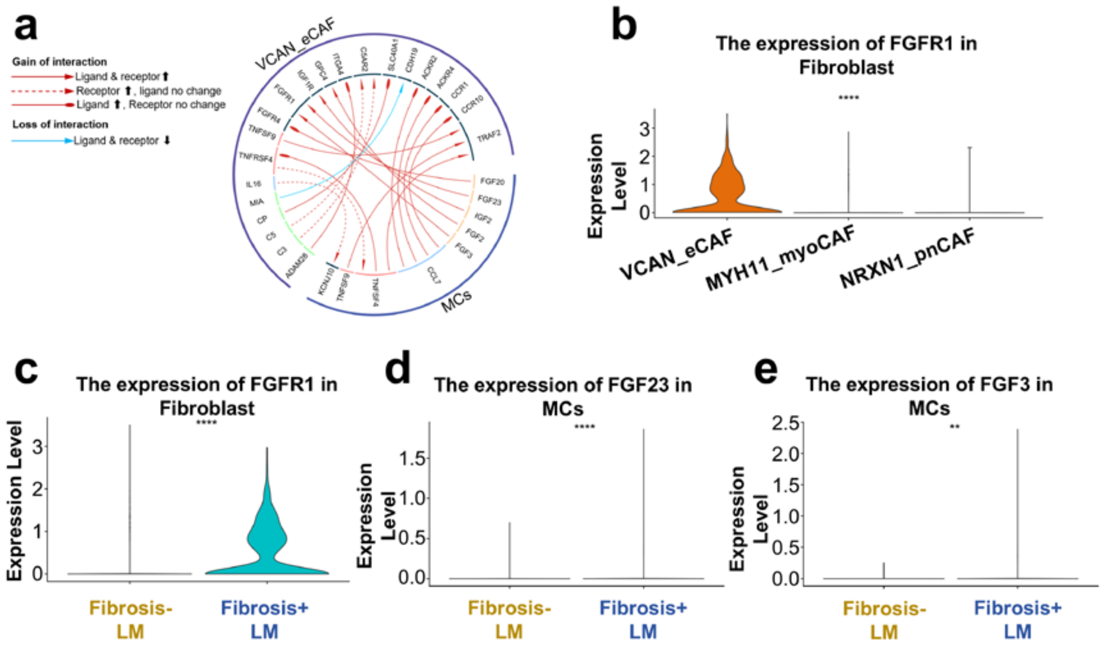

Supplementary Fig. 19. Additional analysis of cell-cell communication between MCs and VCAN_eCAF **a** The significantly upregulated or downregulated ligand-receptor pairs in all signaling pathways between VCAN_eCAF and MCs, estimated by iTalk. **b** The violin plot showing the expression of FGFR1 across CAF clusters, Wilcox rank-sum test, *P* value: ****≤0.0001. **c** The violin plot depicting the expression of FGFR1 in CAFs between the Fibrosis+ LM and Fibrosis- LM, Wilcox rank-sum test, *P* value: ****≤0.0001. **d** The violin plot depicting the expression of FGF23 in MCs between the Fibrosis+ LM and Fibrosis- LM, Wilcox rank-sum test, *P* value: ****≤0.0001. **e** The violin plot depicting the expression of FGF3 in MCs between the Fibrosis+ LM and Fibrosis- LM, Wilcox rank-sum test, *P* value: **≤0.01.
